# Fluorescence Molecular Tomography: Principles and Potential for Pharmaceutical Research

**DOI:** 10.3390/pharmaceutics3020229

**Published:** 2011-04-26

**Authors:** Florian Stuker, Jorge Ripoll, Markus Rudin

**Affiliations:** 1 Institute for Biomedical Engineering, University and ETH Zurich, Wolfgang-Pauli-Strasse 10, 8093 Zurich, Switzerland; 2 Institute of Electronic Structure and Laser - FORTH, Vassilika Vouton, 71110 Heraklion, Greece; 3 Institute of Pharmacology and Toxicology, University Zurich, Winterthurerstrasse 190, 8057 Zurich, Switzerland

**Keywords:** fluorescence molecular tomography, biomedical imaging, optical tomography, fluorescence, hybrid imaging

## Abstract

Fluorescence microscopic imaging is widely used in biomedical research to study molecular and cellular processes in cell culture or tissue samples. This is motivated by the high inherent sensitivity of fluorescence techniques, the spatial resolution that compares favorably with cellular dimensions, the stability of the fluorescent labels used and the sophisticated labeling strategies that have been developed for selectively labeling target molecules. More recently, two and three-dimensional optical imaging methods have also been applied to monitor biological processes in intact biological organisms such as animals or even humans. These whole body optical imaging approaches have to cope with the fact that biological tissue is a highly scattering and absorbing medium. As a consequence, light propagation in tissue is well described by a diffusion approximation and accurate reconstruction of spatial information is demanding. While *in vivo* optical imaging is a highly sensitive method, the signal is strongly surface weighted, *i.e.*, the signal detected from the same light source will become weaker the deeper it is embedded in tissue, and strongly depends on the optical properties of the surrounding tissue. Derivation of quantitative information, therefore, requires tomographic techniques such as fluorescence molecular tomography (FMT), which maps the three-dimensional distribution of a fluorescent probe or protein concentration. The combination of FMT with a structural imaging method such as X-ray computed tomography (CT) or Magnetic Resonance Imaging (MRI) will allow mapping molecular information on a high definition anatomical reference and enable the use of prior information on tissue's optical properties to enhance both resolution and sensitivity. Today many of the fluorescent assays originally developed for studies in cellular systems have been successfully translated for experimental studies in animals. The opportunity of monitoring molecular processes non-invasively in the intact organism is highly attractive from a diagnostic point of view but even more so for the drug developer, who can use the techniques for proof-of-mechanism and proof-of-efficacy studies. This review shall elucidate the current status and potential of fluorescence tomography including recent advances in multimodality imaging approaches for preclinical and clinical drug development.

## 1. Fluorescence Imaging as Tool to Study Molecular Processes

Optical imaging and in particular fluorescence imaging is nowadays widely used for preclinical molecular imaging due to a number of reasons. Optical imaging techniques are sensitive and therefore well suited to detect low concentration of target molecules in tissue. Optical reporter systems such as fluorescent dyes or bioluminescence or fluorescent reporter proteins are stable molecules and can be easily targeted to report on specific molecular processes *in vivo*. Thus optical imaging can build on a wealth of tools that have been developed for optical microscopy. Additionally, the techniques are relatively inexpensive, which facilitates their protrusion into the biomedical research community.

Optical imaging in small animals was originally confined to qualitative or semi-quantitative two dimensional imaging of light distribution on the surface of the specimen, what we shall term here “planar imaging”. The experimental setup for such measurements is simple and consists of wide field illumination for excitation (in the case of fluorescence imaging) and a highly sensitive detector for capturing the fluorescence signals using appropriate filters. Proper quantification is prevented by the fact that light transport in tissue is non-linear, hence the fluorescence intensity measured at the surface cannot be unambiguously assigned to a distribution of fluorescent molecules within the object. For example, it is not possible from a single measurement of light distribution on the surface of a turbid sample to discriminate a focalized, deeply embedded, highly concentrated probe from a disperse weak probe located close to the surface. In order to derive quantitative information from optical imaging data set, two criteria have to be fulfilled: (1) Light transport in tissue has to be accurately accounted for, which requires a theoretical model and adequate knowledge of optical parameters of tissue (absorption and scattering coefficients) and (2) most importantly sufficient number of independent experimental data has to be gathered to allow extracting the unknown distribution of reporter probe within tissue. Progress in understanding and developing models for describing the light/tissue interaction allowed constructing a framework towards three-dimensional quantitative fluorescence tomography.

The number of independent data samples is given by the number of light sources (the different position of the light source tightly focused on the surface) and the number of detector used (source-detector pairs) for each measured excitation/emission set. In the planar imaging mode, wide field illumination would correspond to an infinite number of point sources used simultaneously; yet it is mathematically not possible to deconvolve the contribution of individual sources, preventing reconstruction of the three-dimensional probe distribution. By replacing wide field illumination by a sequential scan of focal light sources, it is possible to measure each individual source detector-pairs at the expense of a prolonged measurement time. This is the basic principle of fluorescence molecular tomography (FMT) [1,2]. Correspondingly, the imaging equipment used for FMT comprises a sensitive (array) detector and a narrow excitation beam, which is scanned point by point over the region of interest using a scanning device. While the first systems described were liquid based and the sample had to be forced into a regular geometrical configuration of an index-matching fluid filled cylinder or a parallel chamber, advances in the theoretical framework and in modeling capacities led to the introduction of so-called non-contact (free space) scanners capable of handling data acquired from arbitrarily shaped objects without the need of matching fluid. Due to difficulties in experimental handling of liquid based systems and the non-natural upright positioning of the animal, non-contact systems allowing horizontal sample positions are more convenient. Nevertheless, liquid based setups offer high sensitivity and high signal-to-noise ratios compared to modern non-contact systems.

Today, FMT provides similar molecular and functional information as radionuclide based positron emission tomography (PET); both modalities suffer from relatively poor spatial resolution in comparison to structural/anatomical imaging modalities such as X-ray computed tomography (CT) and magnetic resonance imaging (MRI), which may render allocation of molecular data to a specific anatomical structure difficult. Another concern related to FMT is the limited penetration depth of light into tissue, which ranges from a few millimeters for wavelengths shorter than 500 nm (green, blue) to several centimeters for wavelengths longer than 650 nm (red, near-infrared). Therefore, FMT is mainly used in combination with probes absorbing and fluorescing in the red to near infrared (NIR) spectral range, for which the light penetration depth is sufficient for pre-clinical imaging using small animals and even humans.

An attractive possibility to account for limited spatial resolution is to combine low resolution molecular imaging techniques (PET or FMT) with CT or MRI, enabling the annotation of anatomical structures with molecular information. In addition, structural information may be used to better confine the reconstruction problem for the low resolution technique. In the case of FMT, structural information together with optical parameters for specific tissues may be incorporated into the forward problem as *a priori* information, which is either acquired sequentially or simultaneously, the latter requiring hybrid imaging instrumentation. Hybrid FMT techniques will become increasingly important in pre-clinical applications as it provides complementary readout of improved quality.

FMT and related techniques such as diffuse optical tomography (DOT), which measure intrinsic tissue properties related to the principal absorbers hemoglobin and deoxyhemoglobin [3], are non-invasive and essentially translatable into the clinics. Established applications of DOT comprise breast imaging, neonatal brain imaging and examination of finger joint in osteoarthritis, amongst others. DOT systems for breast imaging in clinics are even commercially available (ART Advanced Research Technologies Inc., CA and Imaging Diagnostic Systems Inc., US). However, clinical application of FMT is currently hampered by the fact that there are hardly any fluorescent dyes approved for clinical use. As an example, only indocyanine green (ICG) for studies in female breast [4,5] and fluorescein used in ophthalmology [6] have been reported but these fluorescent probes are unspecific, yielding information only on vasculature with very high background fluorescence.

This review focuses on the development of FMT and related technologies and their potential applications in biomedical research and drug discovery. We will briefly discuss the theoretical foundation underlying light propagation in tissue, which is relevant for FMT. Then the development of FMT will be reviewed, with a particular focus on recent technical developments towards hybrid imaging systems. Representative applications of optical imaging in pharmaceutical research illustrating the potential but also the limitations of the approach will be discussed.

## 2. *In vivo* Fluorescence Imaging

Reconstructing the three-dimensional distribution of optical parameters (tissue absorption coefficient, scattering coefficient, fluorophore distribution) comprises three steps: (i) A model of light transport in tissue has to be developed. This model comprises a structural model of the sample itself, which in the simplest approximation consists of a single homogeneous compartment characterized by one set of optical parameters. More realistic tissue models are based on the actual anatomy with different optical parameters for each organ/tissue. (ii) The model is then used to compute the propagation of light emanating from a specific source location throughout the object examined. This so-called forward problem allows estimating the intensity distribution at the sample surface (simulated measurements). For each source location a different surface intensity pattern is obtained. It yields the sensitivity matrix (Jacobian- or weight-matrix), which relates the measurements at the surface to the internal optical properties of the sample. Each element of the sensitivity matrix corresponds to a specific source-detector pair. (iii) The unknown three-dimensional distribution of optical parameters is then estimated from the measurements recorded for the different source locations, the so-called inverse problem. It formally corresponds to inverting the sensitivity matrix. Since scattering dominates light propagation in tissue, light diffuses almost isotropically through the entire sample volume. Each detector element therefore collects information from the whole volume, which renders deconvolution difficult. Typically this ill-posed, under-determined inverse problem cannot be solved analytically but rather by inverting an under-determined matrix through regularization or iterative methods, both being computationally intense. In the following we will outline the most relevant aspects of reconstructing three-dimensional optical data sets.

### 2.1. Light Propagation in Turbid Media

**Figure 1.**
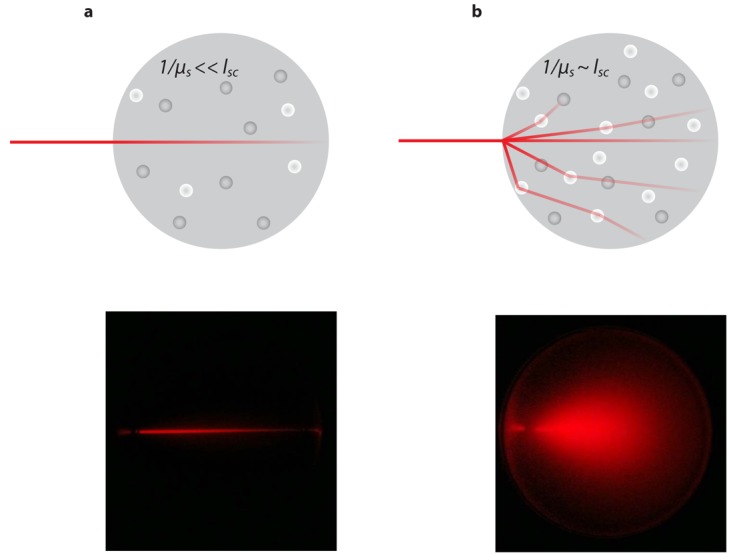
Light propagation in tissue: (**a**), Highly absorbing medium where the mean free path *l_sc_* defining the distance between two scattering events is large compared to 1/*μ_s_*, *μ_s_* being the scattering coefficient. In this case the light intensity decreases with the distance *d* according to *I* ∝ *I*_0_exp(−*μ_t_d*) where *μ_t_* = *μ_s_* + *μ_a_*. (**b**), Highly scattering medium where *l_sc_* is approximately 1/*μ_s_*. This case represents the situation in tissue where light propagation can be modeled with diffusion theory.

Tissue is a highly scattering medium in which light propagates qualitatively as it does in milk. Light propagation in tissue is governed by scattering and absorption events characterized by scattering and absorption coefficient, which depend on the specific tissue type and on the wavelength of the light. The main absorbers in biological tissue are blood in the visual domain of the spectrum and water in the infrared domain. For example, the absorption coefficient of high blood content organs such as the liver, spleen and heart is very high for wavelengths *λ* < 600 nm. During light propagation in tissue light is multiple times deflected from its original direction, with the result that after a short distance (typically 1 mm) light propagates on average in all directions in space (isotropic scattering) leading to a rapid decrease in light intensity with increasing distance from the light source, following the diffusion approximation. Therefore light propagation in tissue cannot be described using a straight ray geometry (Figure 1a) but rather as the migration of an ensemble of light paths that are randomly scattered (Figure 1b). This is achieved by the linear transport theory in turbid medium: conservation of radiance *L*(**r**,**s**,*t*) [Wm^−2^ sr^−1^ s^−1^], which is the amount of light that passes through a particular area at position **r** in a specific direction **s** within a solid angle *d*Ω, is described by the so-called radiative transport equation (RTE) [7]. The RTE can be written as

(1) $$\frac{1}{\upsilon }\frac{\partial L(\text{r},\text{s},t)}{\partial t}+\text{s}\cdot \nabla L(\text{r},\text{s},t)+\mu_{t}L(\text{r},\text{s},t)=\mu_{s}\int_{4\pi }L(\text{r},\text{s}\prime ,t)p(\text{s},\text{s}\prime )d\Omega \prime +S(\text{r},\text{s},t)$$

where *υ* is the speed of light in the medium, *μ_t_* = *μ_a_* + *μ_s_* is the total attenuation coefficient accounting for absorption and scattering and *p*(**s***,***s**′) is the normalized scattering phase function which predicts how light incoming from direction **s**′ is deflected towards direction **s**. The left hand side of Equation (1) describes the effects reducing the total radiance in a certain point **r** in space in a specific direction **s** and the right hand side accounts for the increasing effects.

Solutions of Equation (1) are computationally expensive even for simple conditions/geometries. Therefore for practical applications the diffusion approximation (DA) to the RTE is used providing the advantage of its relative mathematical simplicity. The DA assumes that all contributions to light transport within the medium are from multiple-scattered light and thus the intensity can be considered as diffuse. Furthermore, the DA involves the expansion of the key parameters into spherical harmonics [8] maintaining the linear terms only. The diffusion equation describing light propagation in tissue can be read for a homogeneous medium as

(2) $$\frac{1}{\upsilon }\frac{\partial }{\partial t}U(\text{r},t)-D\nabla^{2}U(\text{r},t)+\mu_{a}U(\text{r},t)=S(\text{r},t)$$

*U*(**r**,*t*) is the average intensity [Wcm^−2^s^−1^] and *D* = 1/3(*μ_a_* + *μ*′*_s_*) the diffusion coefficient [cm] with the reduced scattering coefficient *μ′_s_* = *μ_s_*(1 − *g*), *g* accounting for the anisotropy of scattering (it represents the average cosine of the scattering angle). Note that the radiance has been replaced by the average intensity, *i.e*. in Equation (2) the quantity that obeys the diffusion equation is the average intensity or equivalent, the energy density (*U*/*υ*).

However, one should realize that the assumptions involved start to deviate in distances in the order of the mean free path *l_sc_* from light sources or non-diffusive regions to mention the most relevant constraints. The mean free path is defined as *l_sc_* = 1/*μ_s_* characterizing the distance between two scattering events. For such cases higher order approximations to the RTE are required.

### 2.2. Forward Problem

The forward problem aims at solving the diffusion equation Equation (2) for a specific sample geometry. First concerns about a theory breakdown for sources and detectors separation of less than a few mean free path length [9] was later abolished by showing that even smaller separation can provide good results [10]. For a fluorescent sample embedded in tissue the fluorescence average intensity *U_fl_* excited with an appropriate continuous wave (CW) source located at **r***_s_* and detected by the detector at position **r***_d_* can be described by a volume integral [11,12]

(3) $$U_{fl}({\text{r}}_{s},{\text{r}}_{d})=\int_{V}d^{3}rQ_{\text{instr}}\frac{1}{D^{\lambda_{\text{exc}}}}G^{\lambda_{\text{exc}}}(\text{r},{\text{r}}_{s})\frac{n(\text{r})}{D^{\lambda_{\text{fl}}}}G^{\lambda_{\text{fl}}}({\text{r}}_{d},\text{r})$$

where *Q*_instr_ is a scaling factor accounting for specific parameters of the instrumentation used, *D^λ_fl_^* and *D^λexc^* are the diffusion coefficients at fluorescence and excitation wavelength, respectively, *n*(**r**) is the unknown spatial distribution of fluorochrome concentration. The Green's functions *G^λexc^* (**r**,**r***_s_*) and *G^λ_fl_^*(**r***_d_*,**r**) describe the propagation of light from the point source to a point within the fluorochrome and from a point within the fluorochrome to a point on the detector, respectively (note that in this formula sources and detectors are considered delta functions: if dimensions of these wish to be included, Equation (3) needs to include an integral on the detector and source areas). These functions are related to the structural geometry of the model and there exists different approaches to derive the Green's functions: Analytical solutions only exist for simple geometries such as infinite or semi infinite media [13], slabs [14,15], layered slabs [16,17], parallelepiped [18], cylinder [19], layered cylinders [20] spherical perturbations [21] a summary of solutions for different geometries can also be found in [22]. Although the range of solutions was extended to arbitrary complex shaped geometries based on analytical solutions of the diffusion equation [23]. An aspect to consider, in particular in structured samples, is light propagation at tissue boundaries. For the discussion of boundary conditions we refer to the literature [13]. Alternative strategies to solve Equation (2) have been used, such as Monte Carlo simulations. A detailed summary and references to forward modeling techniques are given in the review of Gibson *et al.* [24].

The use of the linearized Equation (3) is difficult since the instrumental parameter *Q*_instr_ has to be explicitly determined. To overcome this issue Ntziachristos *et al.* [25] developed the concept of normalized measurements, which is based on the ratio of intensities measured at the fluorescence wavelength divided by those measured at the excitation wavelength. The authors demonstrated its feasibility even within the Born approximation, what the authors termed normalized Born approximation. Assuming *Q*_instr_ to be largely wavelength independent, this normalized ratio becomes independent of any instrumental pecularities and was shown to yield robust and accurate data for the distribution of a fluorophore in heterogeneous media [26,27].

The excitation field can be modeled as follows [12]

(4) $$U_{\text{exc}}({\text{r}}_{s},{\text{r}}_{d})=Q_{\text{instr}}\frac{1}{D^{\lambda_{\text{exc}}}}G^{\lambda_{\text{exc}}}({\text{r}}_{d},{\text{r}}_{s})$$

yielding for the normalized average intensity *U^n^*

(5) $$U^{n}({\text{r}}_{s},{\text{r}}_{d})=\frac{U_{\text{fl}}({\text{r}}_{s},{\text{r}}_{d})}{U_{\text{exc}}({\text{r}}_{s},{\text{r}}_{d})}=\frac{1}{D^{\lambda_{\text{fl}}}}\frac{f_{V}d^{3}rG^{\lambda_{\text{exc}}}(\text{r},{\text{r}}_{s})n(\text{r})G^{\lambda_{\text{fl}}}(\text{r},{\text{r}}_{s})}{G^{\lambda_{\text{exc}}}({\text{r}}_{d},{\text{r}}_{s})}$$

For reconstruction purposes Equation (5) can be discretized into *N* volume elements also known as voxels replacing the integration by a summation, which is the Born approximation. For multiple source detector pairs a set of linear equations arises and the forward problem can be written in a matrix form

(6) $${\text{U}}_{M\times 1}={\text{W}}_{M\times N}\cdot {\text{n}}_{N\times 1}$$

where **U***_M_*_×1_ is the measurement vector, **W** weight matrix of the dimension *M* × *N* where *M* is the number of measurements and *N* is the number of Voxels and **n***_N_*_×1_ is the unknown fluorophore concentration distribution in each voxel.

### 2.3. Inverse Problem

Direct inversion of Equation (6) to solve for the unknown parameter **n** is not possible due to the ill-posed (violation of at least one of the properties defined by Hadamard for well-posed problems: A solution exists, the solution is unique and the solution continuously depends on the data), ill-conditioned (a small error in **U** may cause a large error in **n**) and underdetermined (more unknowns than equations) nature of the problem. Standard linear reconstruction techniques such as algebraic methods can be used to solve the problem. Among those the algebraic reconstruction technique (ART) and the simultaneous iterative reconstruction technique (SIRT) [28] are the most common ones. Recently other strategies such as regularization methods (e.g., Tikhonov regularization) for data reconstruction have been proposed which under certain conditions have yielded superior results as compared to ART [29].

Different strategies to solve the inverse problem are iterative procedures: An estimate of the fluorochrome distribution can be found by least-square minimization of the difference between the measured average intensity *U* and the intensity predicted by the forward model *Û*. A detailed description of solution techniques for the inverse problem is beyond the scope of this review article and the interested reader is referred to the literature [8,30].

### 2.4. Fluorescence Probes

Target specific probes that report on cellular and molecular processes in the living organism are the key to molecular imaging. There are two strategies for probe design: exogenous synthetic and genetically encoded reporter systems. The design of exogenous probe comprise a targeting moiety (e.g., a receptor binding ligand) and a reporter moiety that will generate a signal detected by the respective imaging modality. It is important that the reporter group has no or only a minimal effect on the target interaction of the probe, *i.e.*, it should not interfere with the pharmacophor of the targeting moiety responsible for the receptor binding. Molecular targets typically occur at low concentration increasing the demands on the sensitivity of the reporter principle. Optical and in particular fluorescence techniques provide excellent sensitivity and are therefore highly attractive for such applications.

Optical reporters can be divided into three main groups: small synthetic, organic dyes such as cyanine-, oxazine-compounds, genetically encoded fluorescent proteins such as green fluorescent protein (GFP) and semiconductor solid-state nanoparticles such as quantum dots. Dye molecules and fluorescent proteins contain a structure with delocalized *π*-electrons, the dimension of which determines the absorption/excitation spectrum. As a rule of thumb we may state that the longer the *π*-system the more red-shifted the spectrum. This property has been used for the design of red and NIR dyes as well as for the genetical engineering of red-shifted fluorescent proteins [31,32].

Quantum dots display a broad excitation but a narrow emission spectrum, the central frequency of which depends on the physical dimension of the quantum dot (as long it does not exceed the Bohr radius of the electron-hole pair). As a consequence different quantum dots can be excited with a single excitation wavelength, a property that lends itself for multiplexing studies, *i.e.*, simultaneous probing of multiple processes. The drawback of this class of probes is the challenging spectral unmixing procedure to assign the different fluorescent wavelength to the studied multiple processes.

Another interesting class of nanoparticles are phosphor nanoparticles consisting of a crystalline matrix doped with lanthanide ions. They display lower toxicity than quantum dots and offer the possibility of upconversion, which means that emission is observed at a shorter wavelength (*i.e.*, corresponding to a higher energy difference) than the excitation wavelength. The main advantage of these probes is the complete removal of autofluorescence and therefore better imaging performance. The mechanism underlying upconversion is based on multiple photon absorption and shows advantages of sharp emission lines, long lifetimes and superior photostability [33–35]. Phosphor nanoparticles have also been used for X-ray luminescence computed tomography (XLCT) [36,37] which is based on the conversion of X-ray into visible light.

**Figure 2.**
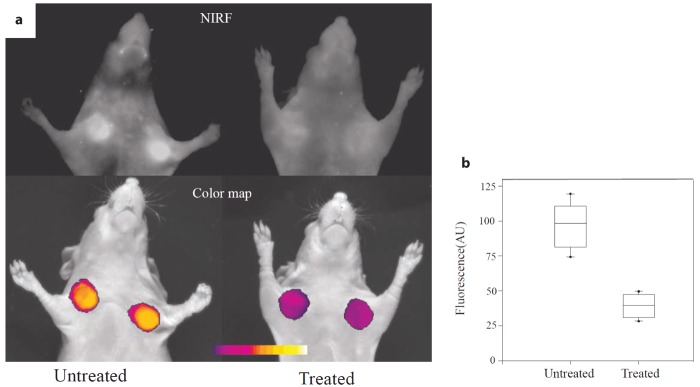
*In vivo* planar imaging of an activatable probe. (**a**), *In vivo* NIR fluorescence imaging of HT1080 tumor-bearing animals. The top row shows raw image acquisition obtained at 700nm emission. Untreated (left), treated with 150 mg/kg prinomastat, twice a day, i.p. for 2 d (right). The bottom row shows color-coded tumoral maps of MMP-2 activity superimposed onto white-light images (no treatment (left), prinomastat treatment (right)). (**b**), Quantitative image analysis of all 20 tumors. Tumoral NIR fluorescence signals are shown in a box plot (bars indicate 10^th^ and 90^th^ percentile). The difference in imaging signal between the two groups was statistically significant (P < 0.0001) (Adapted from [38]; reprinted with permission).

An attractive feature of fluorescence is that it can be modulated by the environment (this, of course, if not taken into account also represents an uncontrolled variable in an imaging experiment). Chemical reactions may modify the structure of the fluorophore and thereby the absorption/emission spectrum of fluorescent dyes may interact with nearby molecules leading to fluorescence quenching or resonance energy transfer. These properties have prompted the development of so-called smart or activatable probes (Figure 2), the fluorescence intensity of which is altered upon interaction with their molecular target, e.g., switched “on” and “off” when the dye is interacting with a nearby quenching molecule [39]. Such probe designs are advantageous since an unbound probe would not give rise to a fluorescence signal, which results in a high target-to-background ratio (TBR). Similar designs are not feasible for radiotracer probe, which are always “on”, *i.e.*, the radionuclide decays with a certain probability; correspondingly TBR may be compromised.

Alternatively, fluorescence light might be generated by bioluminescence. Bioluminescence is a naturally occurring form of chemiluminescence: A (bio)chemical reaction yields a molecular product in an excited state that is stabilized by emission of light. This type of assay is widely applied in biomedical research to visualize molecular processes; in the vast majority of cases oxidases such as firefly luciferase, which generate light upon oxidation of their substrates, are used as a reporter systems.

A detailed description of optical probe design and synthesis is beyond the scope of this review article and the interested reader is referred to the literature [40–43].

## 3. Non-Invasive Imaging Approaches

### 3.1. Planar Imaging

**Figure 3.**
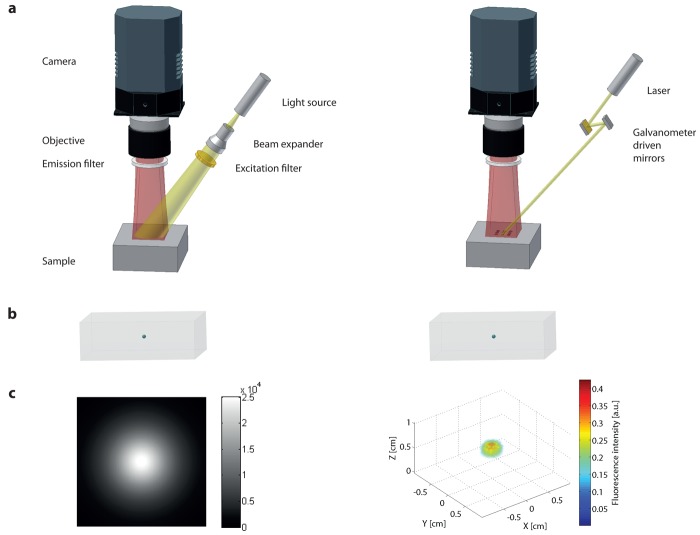
Planar and FMT imaging: (**a**), Setup. Schematic of a planar (left) and an FMT (right) measurement setup and its components in reflection geometry. Transmission geometry (not shown) can be easily achieved by installing either the camera or the source on the other side of the sample. (**b**), Schematic of an embedded fluorophore (turquoise) in depth of a cubic sample (gray). (**c**), (left) Imaging result of the planar setup showing the light distribution at the surface of the sample. Only qualitative information is obtained. (right) Reconstructed fluorophore of an FMT data set allowing the depth resolution and therefore quantification.

The simplest approach for imaging the distribution of a fluorophore *in vivo* is planar imaging, also called fluorescence reflectance imaging (FRI). In this case the signal detected is heavily surface weighted, *i.e.*, is dominated by the contribution of dye molecules at or close to the surface. The instrumental design is such that large parts of the animal or the whole animal is simultaneously illuminated, e.g., using an optically expanded laser beam at the excitation wavelength, light emitting diodes or a white light source using appropriate bandpass filters. The fluorescent light from the tissue surface is detected using a high sensitive charged-coupled device (CCD) camera (Figure 3 left column). The noise requirement (e.g., electronic noise which could potentially influence the imaging performance) of the camera for planar imaging is not as stringent since in general the detection sensitivity limit is governed by the background tissue fluorescence (autofluorescence). The use of bandpass filters with high transmission efficiency for a narrow wavelength range is essential to prevent bleed-through of excitation light into the fluorescent images. The use of lenses with a low f-number, *i.e.* large aperture, warrants a high light collection efficiency. For anatomical registration the fluorescence images are superimposed on mouse images typically obtained with white light illumination. FRI approaches were first described in 1999 [44] and then successfully used to study epidermal growth factor receptor (EGFR) labeled with Cy5.5 in breast tumors [45], tumor protease activity of breast carcinoma [44,46], protease activity in arthritis [47], osteoblast activity in vertebra [48] and GFP expressing tumors [49] in mice.

Fluorescence intensity measured at the detector depends on the length of the total light path (excitation and fluorescence light) through tissue. For FRI fluorescent sources close to the surface contribute much more than deeply embedded ones. In particular autofluorescence of superficial structures will limit the depth field of view of FRI. In comparison, when using transillumination, the total path length is approximately constant irrespective of the location of the fluorescence source within the sample, which enhances the detection probability for deeply embedded fluorescent sources. Fluorescence transillumination was used in various research fields [50] and can be considered as an extension of the transillumination approach used in 1929 by Cutler [51] to resolve absorbing lesions in breast tissue. This method produced shadows attributed to vascular structure (shadowgrams) and has been further developed for breast imaging.

Normalization fluorescence planar imaging in reflectance and transillumination mode has been shown to further improve image quality, depth sensitivity and imaging accuracy compared to non-normalized data [52]. An extension of this imaging approach is the use of spectral information in the image to discriminate the contributions by different fluorochromes or to reduce the contribution of tissue autofluorescence (non-specific background fluorescence) [53,54]. Even though these suggested image improvements makes planar imaging a reliable tool for researchers, it still suffers from a lack in depth resolution and difficult quantification.

In summary planar optical imaging is attractive for qualitative high-throughput measurements demonstrating the presence of a fluorescent probe *in vivo* or in excised tissues. The method provides no depth resolution and correspondingly difficult quantification of the data. Technical simplicity and easy handling makes it an attractive screening modality in the biology lab. In addition planar optical imaging instrumentation is relatively inexpensive.

### 3.2. Tomography Imaging

In order to reconstruct a three-dimensional data set containing *N_V_* = *N_x_* × *N_y_* × *N_z_* voxels *N* independent measurements (each corresponding to an individual source detector pair) are required. As already stated, planar imaging is mathematically equivalent to having a high number of point sources illuminating simultaneously, which implies that each source-detector pairs cannot be distinguished (deconvolved) individually. By using sequential illumination of the individual sources this problem can be circumvented. This is the basic principle of DOT and FMT: Tissue is sequentially illuminated with an array of light sources and the emanating light is captured with an array of detectors (Figure 3 right column). For each source location, of a total *N_s_* sources being sampled, the light distribution on the sample surface is recorded with each detector of the *N_d_* detector array. A light propagation model for the specific sample is developed and parameterized in terms of unknown scattering and absorption coefficient as a function of position in the tissue. By comparing the model prediction with the experimental intensity measurements of all *N_s_* × *N_d_* source-detector pairs, optimized values for the distribution of scattering and absorption parameters are obtained.

The theory was further extended to not only deal with intrinsic contrast but to also account for fluorescence contrast, termed as fluorescence diffuse optical tomography (fDOT). Fluorescence measurements can be obtained using appropriate filters in front of the detectors. Similar generic tomography principles are used for reconstructing the distribution of a fluorescence agent in the tissue. The use of targeted probes in DOT is what finally granted the access of optical tomography into the molecular imaging world. This technique was originally termed Fluorescence-mediated Molecular Tomography, but is currently referred to simply as Fluorescence Molecular Tomography (FMT) [1]. It represents a novel concept and a complete change of mindset and application targets when compared with DOT and fDOT, even though the physics behind them is equivalent. The main goal of FMT is to obtain molecular information on intact biological organisms.

There exist three basic illumination-detection technology schemes for optical tomography imaging [55,56]: (a) the continuous wave (CW) domain using light of constant intensity; (b) the frequency domain (FD) using light of modulated intensity (typically 100 MHz-1 GHz) and (c) the time-domain (TD) using ultrafast laser pulses (femtosecond to picosecond) [57]. The CW approach is attractive, as low cost illumination sources can be used and an optimal signal-to-noise performance is achieved due to the high stability and low noise characteristic of the light sources and detectors. Nevertheless the major drawback of the method is the difficulty of separating the contributions of absorption and scattering from the signal attenuation and the inability to image fluorescence lifetime. Time domain methods resolve the arrival of light from the laser pulse as a function of time at different positions on the tissue. Information from early arriving photons can be used to improve resolution since they have suffered less scattering events [58,59]. Highly diffusive and thus delayed photons are rejected. TD methods are less sensitive (they record a smaller number of photons) and the instrumentation is noisier than CW systems due to time and intensity fluctuations associated with fast switching electronics and pulsing lasers. In FD technologies the laser intensity is frequency modulated, which allows the analysis of the emanating wave with regard to amplitude and phase shift. This allows measuring the tissue optical properties and the fluorochrome distribution. FD methods are less affected by ambient light than CW and TD methods. To improve the resolution compared to CW a modulation of several hundred MHz or higher is required. FD methods are less robust than CW methods because of the reduced signal-to-noise detection involved in sensing high frequencies. Therefore, for molecular imaging applications, where the signals are in general weak, CW technologies are advantageous because of better signal-to-noise performance [55]. This is comparable to DOT, where mainly endogenous absorption changes (e.g., blood oxygenation changes) and scattering properties of tissue are measured. For DOT application the imaging systems are based on TD or FD methods.

One of the first optical tomography scanners was developed for breast imaging in clinics. The DOT measurement setup worked in TD and was based on a parallel plate geometry [60] where the breast was slightly compressed and could therefore considered as a homogeneous slab during reconstruction. Additionally this development was also the first hybrid approach as in the parallel plates MR coils with fiducial markers were implemented to acquire simultaneous data sets. The first ICG enhanced breast images were in good correlation with the acquired contrast agent enhanced MR images in terms of lesion localization [5]. Some years later, driven by the fast development of fluorescent reporter systems in preclinical research and further theoretical understandings in the field of light propagation the first FMT scanner based on CW techniques and designed for cylindrical geometry for preclinical application was reported by Ntziachristos *et al.* [2]. Optical fibers were arranged in rings around a cylindrical cavity and connecting either detectors or light sources with the imaging chamber. The sample is placed vertically in the chamber, which was filled with a fluid matching the average optical properties of living tissue. A respectable spatial resolution of 3 mm (resolved gap between two capillary tubes) and a detection limit of 1nM of Cy5.5 contained in 100 *μ*l was found in cylindrical phantoms [2] and revealed the potential of using optical tomography techniques to detect fluorescent probes in small animals which was demonstrated by resolving enzymatic activity in brain tumors in mice [1]. This index matching fluid was on one hand used to increase signal-to-noise ratio and on the other hand to simplify theoretical constraints associated with boundary modeling: for the reconstruction the irregular object was assumed having quasi-cylindrical geometry.

Cylindrical fiber based systems using matching liquid based methods suffer from several limitations. Due to geometrical constraints only a limited number of fibers can be used, reducing the number of source-detector pairs sampled. Other limitations are light loss due to coupling between tissue and fibers and restrictions with regard to body regions that can be sampled. It was shown in simulation analysis of a plane parallel geometry that an increased field of view and smaller distances between the detector fibers could improve the resolution [61]. Based on this knowledge a vertical parallel plate imager was developed for clinical breast imaging, where the breast is suspended in a tank filled with matching fluid and one plate was movable to compress tissue [4,62]. The source fiber grid was attached on one side of the imaging chamber and the light was acquired on the other side of the chamber by using a CCD camera focused onto the glass window using a set of imaging lenses. With that, the fiber-optic detector could be replaced and the number of detector locations could be enormously increased allowing a higher resolution. This vertical imaging geometry was then also used for preclinical applications in small animals [63]. These systems offer a higher number of source/detector pairs but still suffer from the limitation of fixed geometries, tissue compression and matching fluid. Using such a device a superiority of FMT over FRI could be demonstrated: In a mouse tumor study protease activity in the tumor could only be accurately visualized in the FMT data [63]. A further modification of a parallel plate imager was shown by Zacharakis *et al.* where the plates were horizontally installed and instead of fiber source illumination a free beam illumination was used in combination with a galvanometric driven mirror scanner for the point grid scanning [64].

A major conceptual step was the introduction of so-called non-contact excitation/detection concepts for irregular geometries in FMT, for which there is no geometrical constraint due to optical fibers and no additional light dispersion due to the use of a fluid filled chamber [65]. This required the development of a theory for solving the problem of light propagation from diffuse media (tissue) into free-space (surrounding air) assuming detectors at positions away from the diffuse medium [66]. Experimental feasibility was demonstrated both in phantom studies [67] and *in vivo* [65]. It was successfully shown that a non-contact setup can be built for sample placements in a more natural horizontal position for lymph nodes [68] and lung tumor [69] imaging instead of the previously mentioned upright sample positioning setups. All of the so far mentioned setups have the limitation that due to a fixed positioning only parts of the animal can be imaged. Therefore a concept based on the rotating gantry principle used in CT was used to image the animal 360°. In such systems the sample is placed vertically in a rotating cylinder and can be transilluminated for 360° [70,71]. A further advantage of these systems is the straight forward implementation of the surface capture by white light projection illumination [72]. Yet another interesting setup used to image tumor bearing mice was proposed by Li *et al.* and is based on a conical mirror design [73]. The sample is place horizontally and is surrounded by the conical mirror allowing on one hand a sample surface extraction and on the other hand a non-contact detection and a free-beam illumination covering the animals surface in a half space. Non-contact FMT appears today as a fast and powerful technique for preclinical applications, is characterized by high sensitivity, yet suffers from relatively poor spatial resolution (1 to 2 mm). Resolution is limited by the physical nature of light propagation in tissue, by the unknown anatomical features of the sample, by the uncertainty regarding optical parameters of tissue and restricted number of independent source-detector measurements. The next step would be to use structural information provided by complementary imaging modalities for better confining the reconstruction problem in FMT.

### 3.3. Multimodality Imaging to Derive Multiplexed Information from Biological Systems

In the last decade the role of optics in multimodality imaging or hybrid imaging combining two or more methods to generate complementary read-outs has become an attractive research area. Multimodal *in vivo* imaging systems enable visualization and quantitative assessment of anatomical, physiological, metabolic and cellular properties of an organism as well as its molecular constituents and/or pathways by exploiting different mechanisms generating an imaging signal. Due to economy of scale and technical complexity research today is focused on combining two individual techniques typically combining structural information with an additional readout. Examples are the combination of an anatomical method such as CT, MRI or ultrasound scanning with optical imaging methods providing cellular and molecular insight. Alternatively, optical imaging could be combined with positron emission tomography (PET) or single photon emission computer tomography (SPECT) both providing functional and/or molecular information.

Two strategies can be pursued for merging information generated by different imaging techniques: (1) Measurements can be carried out sequentially using a sample support that is compatible with several modalities. Proper alignment of images (registration) is carried out by data post-processing using anatomical landmarks of fiducial markers based on either rigid body transformation ignoring any deformation or more advanced non-rigid registration algorithms compensating for movement/deformation of the subject. Such an approach benefits from the full performance of the individual imaging modalities. Its obvious disadvantage is that it precludes the simultaneous study of biological processes and that sample motion during the transfer cannot be excluded. (2) By integrating more than one type of imaging technology into one functional unit these issues are inherently addressed. Such a system would allow for simultaneous data acquisition under identical physiological conditions and also simplifies the registration problem due to the fixed geometries connecting both modalities. The technical challenge for an integrated solution is to maintain the performance level of the individual techniques in the combined system, *i.e.*, avoiding or at least minimizing any potential interference between the systems. For biomedical research such devices would be highly attractive as they would allow for multiplexed time-correlated measurements of biological system in the same physiological or diseased state.

A prerequisite for combining two (or more) functional/molecular imaging approaches is the availability of multimodal probes targeting the same biological process but using different reporter moieties that can then be visualized by different modalities.

This section describes the research efforts under way to combine optical imaging techniques with other imaging modalities. It outlines the benefits of the different combinations to investigate novel therapeutic or diagnostic approaches in animal models of human disease. A list with different hardware based approaches to combine optical imaging techniques with other state-of-the-art methods is given and applications concerning the specific combination are mentioned. To complete, a short insight in multimodal probe design and its applications is included.

#### 3.3.1. Multiplexing Information Using a Sample Support Compatible with Multiple Imaging Modalities

A straightforward approach for multiplexing imaging information is the development of an animal support, which is compatible with the different commercially available imaging modalities. This support ensures a reproducible animal position and included fiducial markers visible by both systems allowing a precise co-registration after the measurements. It is obvious that any combination of imaging modalities is amenable to this approach provided the appropriate fiducial markers are used. The crucial part in such experiments is the subject transport from one to the other modality. Care has to be taken to avoid/minimize animal movements during the transport and to maintain stable physiology e.g., by avoiding changes in body temperature. Alignment of multimodal data is then achieved by post-processing as already described.

The potential of this approach was early demonstrated by combining DOT and functional MRI (fMRI), both methods assessing changes in blood oxygenation, demonstrated similar temporal responses in the somatosensory cortex evoked by electrical forepaw stimulation [74].

**Figure 4.**
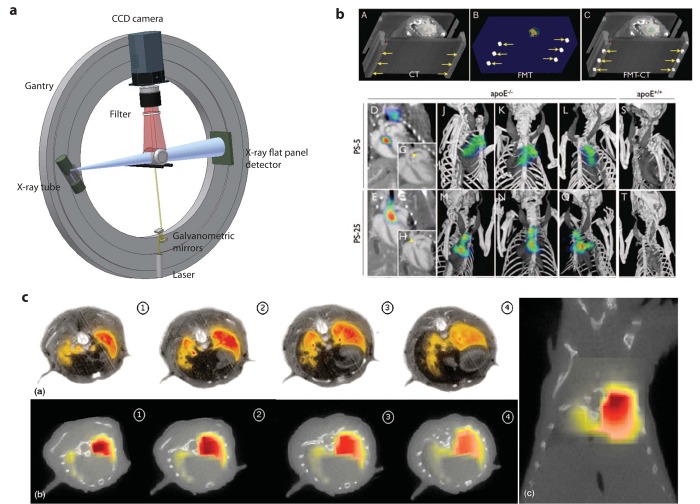
Applications: (**a**), Schematic of a hybrid FMT/CT system. Perpendicular to the X-ray tube and flat panel detector axis the FMT instrumentation is mounted consisting of a laser source, a scanning device and a CCD camera. All components are mounted on a rotating gantry holding the sample in its center.(**b**), Sequential FMT/CT example of artherosclerotic plaques in mice. A through C, Image co-registration is based on fiducial landmarks (arrows) that are incorporated into the animal holder and are identifiable on CT (A) and FMT (B). The software co-aligns these fiducial markers to created a hybrid data set (C). Fluorescence signal in the aortic root of an apoE^−^/^−^ mouse is encircled. D and E, 2-dimensional FMT/CT long axis views of apoE^−^/^−^ mice injected with respective protease sensors. Fluorescence signal is observed in the aortic root and arch, regions with high plaque load. G and H, CT only views of D and E. Arrow heads depict vascular calcification, likely colocalizing with plaques. J through O, 3-dimensional maximum intensity projection of hybrid data sets show skeletal and vascular anatomy and the distribution of fluorescence signal. Most signal is observed in the root and arch. S and T, FMT/CT after injection of respective sensor into wild-type mice (Adapted from [76]; reprinted with permission). (**c**), Hybrid imaging example of a lung inflammation mouse model. (a) Cryoslice images overlaid with normalized fluorescence image; reconstruction using weighted segments regularization (b) transversal slices and (c) coronal slice (Adapted from [82]; reprinted with permission).

**Figure 5.**
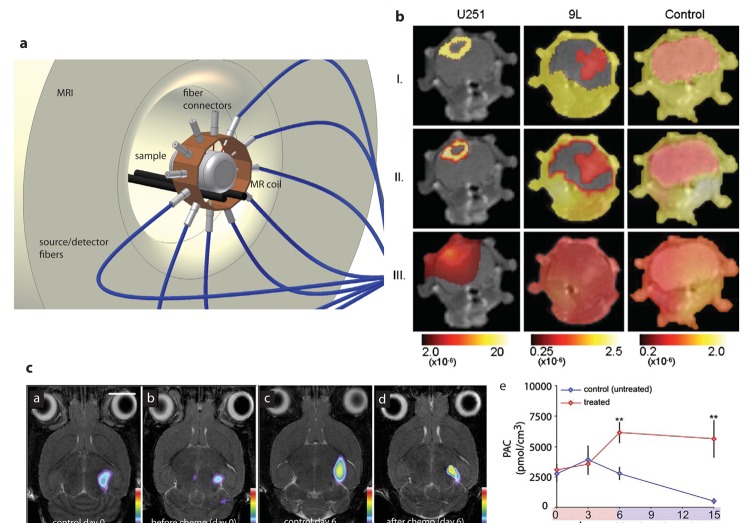
Applications: (**a**), Schematic FMT insert for a clinical MRI system. The system consists of source/detector fibers which are mounted circularly around the sample and which were fixed in the MR coil. The detector and the sources are located outside the magnetic field (**b**). Example of a simultaneously acquired data set with a setup presented in (a). Representative images of fluorescence yield overlying the corresponding MR images. Each column provides results for a single animal from one of the three mouse populations (U251 tumors, 9 L tumors, and controls, respectively). The rows show images recovered using the hard-prior technique (I), the soft-prior technique (II), and an unguided reconstruction technique (III) (Adapted from [103]; reprinted with permission). (**c**) Example of a sequentially acquired data set showing that chemotherapy increases the ratio of protease activity to tumor volume. (a), (c) Combined FMT/MR imaging of a mouse bearing an implanted glioma at day 0 and day 6. (b), (d) Combined FMT/MR imaging of a chemotherapy treated mouse bearing a similarly sized glioma at day 0 and 6. (**e**) Quantification of the effects of chemotherapy on protease activity and tumor size. Protease activity concentration (PAC) was significantly altered by the end of a 5 day course of chemotherapy. This difference became even more pronounced at the end of the experiment (day 15 after chemotherapy). (Adapted from [75]; reprinted with permission).

The first study combining FMT with MRI was carried out by Ntziachristos *et al.* [1] who analyzed cathepsin B activation in 9L gliosarcoma in nude mice. By superposition of the reconstructed fluorescence intensity distribution indicating protease activity and the gadolinium enhanced MR image the correct localization of the optical signal in the corresponding MR brain slice could be revealed. A related study of a mouse brain glioma model was recently performed by McCann *et al.* [75] where FMT was applied to image protease activity in the tumor using a commercial FMT system while the subsequently recorded MRI data provided the anatomical reference. The authors claim that only the combined analysis of FMT and MRI data correctly predicted the effects of chemotherapeutic intervention (Figure 5c) by calculating the ratio of ProSense fluorescence (extracted from FMT) to tumor volume (extracted from MRI), yielding a normalized protease activity which is here called protease activity concentration (PAC). Another example was the protease activatable fluorescence probes designed to image atherosclerosis in mice [76]. Due to the small size of atherosclerotic plaques and the variability in their distribution across the vascular system, accurate localization of sites of protease activity with FMT is challenging. To overcome this limitation a multimodal imaging cartridge that holds the mouse and is compatible with both FMT and CT has been used. The mouse is slightly compressed by two optically translucent windows in the cartridge preventing animal motion during transfer between the modalities. In this work it was shown that FMT enables visualization of protease activity at plaque sites as well as the effect of therapeutic interventions reducing plaque associated inflammatory processes in a longitudinal manner. Registration of imaging data in the course of the study has been warranted on the basis of a high resolution contrast-enhanced CT angiography, which provided anatomical landmarks [76] (Figure 4b).

By having a MR sample platform which is compatible with a bioluminescence system, it has been shown that the accurate knowledge of tissue layers segmented from an MR image can improve the bioluminescence imaging [77].

Finally, sequential nuclear-optical imaging using a planar imaging configuration has been demonstrated in tumor bearing mice [78,79]. Selective accumulation of perfusion markers using 99m-technetium (^99^*^m^* Tc) marker as radionuclide emitting gamma photons and hematoporphyrin as a fluorophore, has been demonstrated.

Even tri-modality systems combining CT/PET/FMT were proposed where the animal was positioned on a mobile platform which is shifted on a rail from the PET/CT scanner to an FMT scanner [80]. Other multimodality readouts can be achieved by placing the different systems in close proximity allowing a fast shifting of the animal from one to the other modality. Such a readout to improve the quantification of tumor burden in mice was performed by placing a PET/CT scanner close to a bioluminescence system [81].

#### 3.3.2. Hybrid Systems Combining Structural and Optical Imaging: Annotation of Anatomy with Molecular Information

Even though significant progress in terms of spatial resolution and sensitivity has been recently achieved in the field of FMT, the method is still far from accurately visualizing biological information at spatial resolution comparable to structural imaging modalities such as CT. This lack of accuracy is due to the severe scattering of light in a turbid medium such as tissue, which leads to a loss of spatial information which deteriorates as the distance covered by the light path exceeds the scattering path length (typically 1mm in tissues). Even though the principles of light propagation are well understood, the exact solution of the mathematical models that accurately describe the light distribution inside the imaged volume remains extremely computer intensive. This is among other reasons due to the fact that simple models with direct analytical solutions have difficulties in accounting for tissue heterogeneity (including multiple tissue interfaces of arbitrary geometry), that the optical parameters for the various tissues are known with a large degree of approximation and, more importantly, greatly vary with the physiological state of the tissue (more specifically, it's the oxygenation state of the tissue which causes changes in the absorption properties of tissues-this very fact is the basis of DOT), and that the problem is commonly underdetermined, *i.e.*, the number of independent data samples (*i.e.*, the measurements corresponding to a source detector pair) is insufficient for achieving high spatial resolution. Therefore the FMT reconstruction, which is based on maps representing the two-dimensional light distribution on the surface of the subject, can only be performed by introducing approximations, which in general represents over-simplifications. Another reason is that a hybrid system providing structural information on the anatomy (internal geometry), would help to implement more sophisticated models accounting for the tissue interfaces and for the absorption and scattering properties within each type of tissue.

##### X-ray CT/Optical imaging

X-rays are electromagnetic waves of short wavelength, which interact with the constituents (mainly electrons) of the matter penetrated. Contrast in X-ray imaging is based on the differences in attenuation coefficient of the various tissues. Conventional X-ray images are two-dimensional projections of three dimensional objects; they are widely known as a medical diagnostic tool. They exhibit weak contrast among soft tissues but high contrast between soft tissue and bone and are therefore useful for detecting pathologies of the skeletal system. By using contrast agents, which are electrodense materials, the contrast among soft tissues may be enhanced. The objective of CT is to obtain three-dimensional anatomical information by reconstruction of the distribution of attenuation coefficients. This is achieved by recording multiple projections of the object from various angles and applying reconstruction procedure via back-projection [28]. For representation the three-dimensional data stack is re-sliced to yield two-dimensional cross-sectional images.

A first successful attempt to combine CT with FMT based on sequentially recorded data was reported in 2005 [83]. In this study a parallel plate FMT imager [64] was used to image a cancer model with GFP expressing glioma cells implanted into the lungs of nude mice. For FMT experiments the animals were positioned vertically in a chamber consisting of parallel glass plates, which was filled with refractive index matching fluid. The reconstructed FMT data set was then manually aligned to the corresponding CT data to obtain adequate data co-registration. A major disadvantage of this FMT setup is the animal placement in a liquid filled imaging chamber, which renders studies in the head region of an animal very difficult. Therefore, multimodal sequential imaging setups using a liquid free 360° FMT setup, in which the specimen is placed vertically on a rotating stage have been developed [71,84]. The anatomical reference was in both studies acquired afterwards with a *μ*-CT system. Such a setup was used to visualize the deposition of amyloid-*β* plaques in a murine model of Alzheimer's disease. Data registration was achieved by an affine transformation mapping the animal's surface as obtained using a surface projection technique in the FMT setup [72] with the surface derived from the CT data set. Once registered the CT slices could then be used as anatomical reference for the corresponding optical slice. The plaque load in a transgenic APP23 mouse strain could be visualized following the administration of the NIR dye AOI987 [85], an oxazine dye known to bind selectively to the amyloid plaques in the mouse brain. This hybrid imaging approach allowed monitoring the increase in plaque load during disease progression [86]. Still the vertical posture of the animal for the FMT measurement system is not satisfying due to gravity induced displacement of organs/tissue compared to the horizontal placement in common CT scanners. Therefore it is desirable to mount the optical components on a CT gantry, which rotates around the animal in horizontal posture [87].

A successful implementation of a combined FMT/CT setup with this gantry approach was described by Barber *et al.* [88], who fixed the optical components and the CT components on two rotating gantries side by side on parallel planes. The sample platform is centered in the symmetry axis of the gantries which allowed a translation from the optical to the CT part without removing the platform. A study using cylindrical and arbitrary shaped phantoms with different inclusions containing the fluorescent dye ICG demonstrated the potential of the system for *in vivo* applications. By including information on scattering and absorption parameters derived from intrinsic optical measurements (with DOT) using this setup, the authors could improve the reconstruction quality for the embedded fluorescent object [89]. The suitability of the system for *in vivo* application was recently shown in mouse experiments: Both the location of a superficial and a deep seated fluorescent inclusion could be accurately reconstructed [90]. Further improvements in reconstruction accuracy have been achieved by incorporation of structural information derived from the CT and of the functional background information from DOT.

Another rotating gantry approach with the optical components for the FMT imaging mounted on the CT gantry with the optical axis at a right angle to the axis comprising the X-ray source and detector has been described by Schulz *et al.* [91](Figure 4a). This alignment allows the simultaneous acquisition of both data sets, which are inherently co-registered. Structural information from CT was used to guide the reconstruction of FMT data. The method was used to study focal lesions in the brain [91] and the lungs [82] of mice (Figure 4c). A mixture of 1*μ*Mol/L Alexa750 fluorochrome was implanted stereotactically into the right brain lobe at a depth of 4mm in euthanized mice. The location of the sample could be adequately reconstructed with both modalities. In a further validation study protease activity was assessed in a lung tumor model. A protease sensitive fluorescence probe was tail vein injected 24h prior to imaging. Distinct fluorescence signal has been observed in the lung of the mouse following cleavage of the probe by tumor secreted proteases [92]. Feasibility of accurate localization of lesion in the brain and in lung tumors with help of prior structural information was demonstrated and illustrated the potential future of this application.

The design of combining the free-space FMT with CT on a vertically mounted gantry rotating around a sample is attractive as animals can be scanned in their natural horizontal posture. Yet, the irregular shape of the object renders reconstruction of FMT data challenging. An alternative approach involves an arrangement of FMT and CT sources and detectors in a horizontal plane with the sample rotating around a vertical axis. This would allow immersion into refractive index matching fluid to achieve regular geometry for data reconstruction [93,94]. Feasibility of this design was successfully demonstrated in experiments using standardized tissue phantoms and euthanized animals carrying a esophageal tube filled with a fluorescence dye. Da Silva *et al.* [95] used a similar system for studying lung metastasis in nude mice. *In vivo* measurement were carried out following administration of a transferrin/Alexa 750 conjugate and demonstrated the presence of fluorophore in the lung, liver and bladder overlaid on a structural image showing the animals skeleton for accurate anatomical localization of the fluorophore distribution. A similar setup was built to combine bioluminescent tomography and CT imaging [96]. The authors demonstrated an increased localization and quantification accuracy in bioluminescence tomography for a heterogeneous mouse model incorporating the segmented CT data compared to a standard homogeneous mouse model.

Due to the limited tissue penetration of light, FMT/CT systems are largely confined to preclinical applications, although optical imaging is used for very specific applications in clinics such as breast- and testicular-cancer imaging, arthritis and neonatal brain investigations. Nevertheless, clinical systems have been designed as well. Today, X-ray radiography is the most widely used screening methods in clinics, e.g., in screening for mammary carcinoma or articular cartilage damage in osteoarthritis. Although X-ray provides high resolution in differentiating bones from soft tissue the image contrast between different soft tissues is very low. Therefore, adding functional or molecular information would definitely enhance the diagnostic accuracy. A characteristic of invasive tumor is sustained angiogenesis, which is associated with high microvascular density and potentially also a high tumor blood volume. Hence combining structural imaging with a method that provides hemodynamic information such as DOT, which measures local values of scattering and attenuation coefficients, which are largely governed by the absorbers contained in blood (hemoglobin and oxy-hemoglobin), might be diagnostically relevant.

Therefore, a DOT imaging system compatible with a mammography device has been developed [97,98] for clinical trials. In these studies, the measurements where sequentially recorded without moving the breast. DOT measurements revealed in increased absorption in the tumor region indicative of increased blood volume and alterations in oxygen saturation. The tumor location was verified by X-ray imaging.

The possibility to access the molecular and cellular signature of osteoarthritis by diagnosing cartilage abnormalities and alterations in composition of synovial fluid in joints via absorption and scattering spectra measurements with DOT was shown by Yuan *et al.* [99]. The optical properties in the fluid which is changing depending on the inflammation state is measured with DOT. The low spatial resolution caused by multiple scattering of light was improved by incorporating structural *a priori* information for the optical reconstruction. DOT data quality was such that joint-space abnormality in osteoarthritic joints could clearly be identified [100], highlighting the potential of the multimodal concept for clinical diagnosis and treatment control.

Recently XLCT was proposed as a new combination of structural and molecular readout within the same system [101,102]. It is based on the selective excitation and optical detection of X-ray excitable phosphor nanoparticles. These nanoparticles can be designed using various methods [36,37] to emit NIR light when excited with X-rays and are therefore well suited for biomedical *in vivo* application. Imaging can be performed simultaneously and no further optical light source is needed for excitation, the particles are excited to emit light by high energy photons produced by interaction of X- and *γ*-rays with matter through photoelectric absorption or Compton scatter. The major advantage is the benefit of having no autofluorescence produced by an optical excitation source and that also very few photons detected by the camera are used for a precise fluorophore localization. As this imaging technique is very new, there are currently only proof of principle experiments in phantoms available and a real *in vivo* imaging proof is still missing.

##### MRI/Optical imaging

MR images represent a weighted distribution of hydrogen nuclei (protons) in the body, the principal signal contributors being the protons of water and adipose tissue. Nuclei with odd proton and/or neutron number such as the proton possess a magnetic moment, *i.e.*, behave as tiny magnets. In a strong magnetic field magnetic moments align either parallel or opposed (anti-parallel) to the field direction, with slight excess in the parallel orientation leading to a net macroscopic magnetization. Irradiation with electromagnetic waves of a certain frequency (the Larmor frequency, which corresponds to the difference in energy between the parallel and anti-parallel state) will excite the spin system and generate observable non-equilibrium magnetization that is measured using an MRI receiver coil. The MRI signals decay by relaxation processes (T_1_,T_2_,T_2_*) that are tissue specific and constitute the source of the high soft-tissue contrast of the method. The high magnetic field strengths (>1.5T) and the restricted access to the sample render a hybrid design involving the incorporation of the FMT into an MRI scanner, rather challenging. Non-magnetic optical components have been developed for this purpose.

Instrumentation and theoretical development for combining MRI with optical imaging was driven by the need of an efficient detection for early stage breast tumor in women. Breast tissue is relatively weakly absorbing and therefore lend itself for optical imaging studies. As discussed in the previous section the structural and dynamic contrast enhanced MRI imaging method was complemented by optical imaging targeting tumor angiogenesis. The sensitivity of the optical readout for assessing the tumor vasculature was increased by using the fluorescent dye ICG, an intravascular agent approved for clinical use [5]. An overview about technologies for breast cancer management with optical and combined optical and MR systems is given in [104]. FMT may become a powerful tool in breast imaging once target-specific fluorescent agents capable of highlighting physiological processes associated with cancer pathology become available for diagnostic applications. It is not surprising that breast studies are in the focus of FMT/DOT method development. Experiments using “breast-like” phantoms with fluorescent inclusions [105] or simulations based on MR images of breast tissue [106] should demonstrate the potential of these combined techniques.

Nevertheless, the focus of studies addressing hybrid FMT/MRI systems currently relate to preclinical applications. All solutions found in literature uses a fiber based FMT as an insert into either a clinical MRI magnet or a preclinical MR scanner (see Figure 5a). For this setup the fibers have to be brought in physical contact with the sample. Such design constitutes a compromise in terms of imaging performance for both modalities. Measuring animals using a human scanner with a large bore compared to the animal will compromise spatial resolution and thus the quality of structural information. On the other hand performance is affected by light loss due to inevitable loss in coupling to the fibers guiding the light to the detectors located outside the MRI magnet and that these fibers recover light only from a small part of the surface. Moreover such systems are bulky and restricted with regard to the number of source detector pairs and also the flexibility in potential excitation schemes. Nevertheless such a system was successfully implemented and proof of concept was demonstrated for mouse brain where human glioma tumor cells were implanted intracranially [107,108]. The tumor area was segmented according to the MRI image and an improvement in tumor localization could be shown by applying the prior knowledge. Recently the setup has been used to assess the EGFR status in an animal model of brain tumor. Tissue regions segmented on the basis of the MR image were used to guide the reconstruction of fluorescence distribution of the NIR fluorescent dye conjugated to the EGF ligand [109]. The setup comprises a single ring of fibers connected to light sources and detectors and therefore allows the fluorescence reconstruction of one single slice. The corresponding slice in the MRI data is identified on the basis of fiducial markers mounted at the fiber tip in contact with the animal. Similar systems has been developed for DOT imaging to measure changes to study rat cerebral oxygen consumption [110] or to visualize increased blood volume in rodent breast tumor models [111]. In all cases, structural information was used for the reconstruction of optical data.

Integrated DOT/MRI systems have also been described for clinical applications [5]. For example, hybrid imaging has been used to compare the fMRI signal evoked by an external stimulus with hemodynamic changes assessed by DOT. Consistency in spatial localizations of fMRI and DOT signals in activated brain regions was demonstrated. Structural MRI information has been used to generate a patient specific head model [112] or to fuse fMRI and DOT information into one model to generate a joint estimate of the underlying physiological contrast [113].

##### Ultrasound/Optical imaging

In this section we will not discuss opto-acoustic imaging, in which ultrasonic waves elicited by light absorption are detected. This is a very exciting new modality with great potential both in pre-clinical and clinical studies, but it is out of the scope of this review. For further information on this technique we refer to [114,115]. We will focus on the use of ultrasound imaging providing pure structural information as anatomical reference for FMT images or to improve FMT/DOT localization.

For example, ultrasound images have been used for verifying the correct spatial localization of fluorophore distribution in a phantom mimicking prostate in a study using a newly developed transrectal probe for the fluorescence measurements [116]. The two data sets have been recorded sequentially. Clinical applications were once more driven by the need for improved detection of malignant breast lesions. Structural information derived from ultrasound was used to segment breast tissue into tumor and normal tissue to guide the optical reconstruction [117]. This study was performed by a combined ultrasound/DOT imager [118] where the ultrasound array was centrally placed in a plate comprising the source and detector fibers for DOT imaging. A related study with a further developed instrument was performed by Zhu *et al.* [119]. A recent clinical application deals with image-guided prostate tumor biopsy using an ultrasound/FMT setup. Standard of care in supposed prostate cancer is an ultrasound guided biopsy. It has been speculated that hybrid ultrasound/FMT might improve the accuracy of the biopsy procedure [120]. Phantom experiments with a trans-rectal ultrasound coupled NIR device could show the improved fluorophore localization from the optical reconstruction with help of prior knowledge of ultrasound structural information [121]. The same instrument was used to investigate transmissible venereal tumors *in vivo* in a dog model. Tumor tissue displayed significantly different scattering and absorption coefficients as compared to the normal prostate tissue. There was good agreement in tumor localization between ultrasound images and the reconstructed optical images [122].

#### 3.3.3. Hybrid Systems Combining two Functional/Molecular Imaging Modalities

Combining optical with a second functional imaging modality such as PET, SPECT or functional/physiological MRI (e.g., dynamic contrast enhanced MRI to assess vascular permeability) allows investigating two concurrent biological processes using two distinct target-specific imaging probes or generating multiplexed readouts of the same process using fused probes that contain two reporter moieties. A variety of research applications is given in [123] e.g., cross validation of both methods and the use of both modalities in intraoperative procedures. The hardware development in this field is driven by integrating the two methods into one system allowing the simultaneous data acquisition of a sample, which is critical when correlating functional readouts.

##### SPECT/PET/Optical imaging

An optical/PET detector concept was proposed in 2004 which is capable of efficiently detecting the 511 keV photons emitted from PET radiotracers and to simultaneously measure faint bioluminescence signals from luciferase reporter genes utilizing transparent scintillators [124,125]. The concept [126,127] was successfully tested on the bench with phantom measurements for high energy 511keV and visible low energy 2–3 eV photons [128]. However, *in vivo* proof of principle of this approach is still lacking. Simulations for the light propagation indicated that accurate localization of bioluminescence sources within the simulated phantom mouse should be feasible [129]. Yet, accurate localization required knowledge of the optical properties of the tissues *in vivo* [130] and by spectrally resolved bioluminescence signals, which both appear difficult under *in vivo* conditions. Another set of simulations was carried out to evaluate the PET characteristics of the detector [131]. Apparently, modeling predicts feasibility of the approach yet an *in vivo* application using this combined imaging detector has not been reported. A potential issue with the device might be the so-called afterglow effect, *i.e.*, low energy photons generated by the reaction of high energy photons with the scintillation crystals [132]. These produced low energy photons have similar properties as the photons emanating from the bioluminescence probe and can therefore not be discriminated, which might lead to a misinterpretation of the optical measurements. Depending on the scintillation material used these effects can be reduced to an amount that simultaneous signal read out should be possible.

A more sophisticated hardware development was shown by Peter *et al.* [133] allowing not only bioluminescence but also fluorescence measurements using the same setup. It consists of two different detector rings, an outer PET detector ring and an inner ring comprising optical detectors. The laser light is coupled into the system through the gaps between the detector elements of both rings. First phantom experiments showed that the system is sensitive for detecting micromolar fluorescence dye concentrations and nuclear activity at a microcurie level. Again, *in vivo* validation has not yet been reported.

An attractive role of combined PET/FMT is to validate FMT as a surrogate for the nuclear imaging modality, in particular for preclinical application. The use of FMT would offer several advantages: (1) FMT is significantly cheaper then PET, (2) fluorescent tracer are stable in contrast to positron emitters that are characterized by short half lives and (3) the fluorescent signal can be modulated as seen, e.g., for activatable probes. The major disadvantage of FMT as compared to PET is quantification. Therefore it was important to analyze whether and how well probe concentrations and spatial signal distributions of hybrid PET and optical tracers were correlated: Both phantoms and *in vivo* studies in mice bearing subcutaneous tumor xenografts have been carried out [134]. As an anatomical reference the CT image was used captured with a hybrid PET/CT system. The authors reported high values for the correlation and concluded that FMT in fact could be used as a sensitive and quantitative tool assisting in the development of PET reporters. This question was further addressed by Garofalakis *et al.* who studied deep seated organs like the kidney [135]. A mixture of a fluorophore and a ^18^F labeled oligonucleotide, that has been shown earlier to accumulate in the kidney, was intravenously injected into the mouse prior the PET and FMT measurements. By analyzing the reconstructed PET and the FMT scans with help of an anatomical CT image they found a discrepancy of the geometrical volumes but nevertheless a good correspondence of tracer concentration as determined by the two modalities. This led to the conclusion that free-space FMT is capable of providing information comparable to PET even for deep seated organs, although FMT has some sensitivity limitations compared to PET.

A peculiarity of combining PET imaging with optical imaging is Cherenkov radiation, which can be observed as a side-product of positron emitting radiotracers. The high energy positron emitted by the radioisotope may transiently exceed the speed of light in tissue and therefore satisfies the requirements for generating Cherenkov radiation, *i.e.*, the charged particle induces local polarization along its paths through the medium and radiation is emitted when the excess energy is released and system returns to equilibrium. The Cherenkov radiation has a continuous spectrum from the near ultraviolet to NIR and is emitted at a particular angle (which depends on the particle velocity and the velocity of light in medium). The intensity is inversely proportional to the square of the wavelengths. Cherenkov light was successfully detected in tissue phantoms using optical planar imaging in combination with PET using wells of the radionuclides ^18^F and ^13^N and *in vivo* in a tumor xenograft model using [^18^F]-FDG injection [136]. These results where confirmed by another study using ^18^F radionuclides and further validated using ^131^I and ^90^Y, two widely used isotopes for radiotherapy [137]. The usage of Cherenkov light is very limited to the study of subsurface structures as the emission wavelength and the intensity is much higher in the UV than in the red spectral domain.

### 3.4. Use of Prior Information for DOT/FMT Reconstruction

The achievable image quality and resolution, particularly the spatial resolution is limited by the ill-posed, underdetermined and ill-conditioned nature of imaging with diffuse light [8,30]. Improvements in resolution and imaging performance can be achieved by incorporating anatomical (CT, MRI) or functional (PET) information, so-called image priors in the DOT/FMT reconstruction process.

Tissue classification is achieved by segmentation of anatomical images. This knowledge is then introduced in the reconstruction process by adapting the regularization procedure through the inclusion of a so-called penalty function. The impact of these procedures was recently demonstrated on small animal FMT/CT data sets and simulations in a lung inflammation model [82].

Apart from structural there are functional priors. The diffusion equation contains terms of absorption and scattering which need to be defined to perform the forward modeling. This is referred to functional prior information and can be incorporated in the reconstruction by assigning appropriate optical parameters to anatomical regions in the forward problem. In a paper by Hyde *et al.* [138] the effect of optical properties on the reconstruction quality using priors was examined for a normalized Born approximation. The authors conclude that the prior information in the inverse problem alone can improve the performance and a further enhancement could be achieved by an improved diffusion modeling with average optical properties assigned to the segmented tissue. The value of using functional *a priori* information was demonstrated by DOT guided fluorescence tomography [139] for which an improved distribution of the accurate fluorophore concentration has been computed in phantom study when reconstructing data obtained with a 360° non-contact DOT/FMT system. Others also emphasize the significance of functional *a priori* information [140,141] in terms of localization accuracy. The role of having both functional and structural *a priori* information was studied in multi-compartment phantoms producing a heterogeneous background [142,143]. The heterogeneity in the attenuation coefficients was accounted for when analyzing DOT images acquired prior to the FMT data.

### 3.5. Multimodality Probes

Multimodality probes can simplify simultaneous imaging with hybrid systems due to single probe administration revealing a contrast enhancement on both modalities. A detailed description of the synthesis and production procedure is beyond the scope of this review article. The interested reader is referred to the references in the following mentioned citations. Designing a probe for multimodality imaging is most prominent a combination of optical and radionuclide labeled probe both imaging functionality of a biological process. A tumor study of a human melanoma xenograft model combining a ^111^In chelator diethylene-triamine-pentaacetic acid and a NIR fluorescent dye showed an improved resolution and sensitivity with optical planar imaging for superficial lesions compared to sequentially acquired *γ*-scintigraphy images providing sensitive detection of deeper structures [144]. A similar study combined bioluminescence, fluorescence, *γ*-scintigraphy and SPECT readouts [145]. A complementary imaging strategy for optical and scintigraphic imaging involves the incorporation of and activatable fluorescent molecular system into a radio-labeled cleavable peptide with identical pharmacokinetics but different reporting strategies [146].

An attractive concept is the development of combined probes resulting in contrast enhancements on both modalities. Josephson *et al.* [147] developed an MRI contrast agent based on magnetic nanoparticles with an enzymatic activated NIR fluorophore. This probe can be localized with MRI while providing information on molecular processes (protease activity) by fluorescence imaging techniques. Another dual fluorescent/MRI probe based on liposomes was developed by Shan *et al.* [148] which enhanced on one hand the tumor morphology in the MR images and allowed at the same time the quantification of the biomarker expressed in the tumor using NIR fluorescence optical imaging. Recently the *in vivo* application of an up-converting luminescence probe for optical imaging in combination with a paramagnetic MRI probe enhancing the MR contrast was reported as a future concept to detect and image biological targets in a dualmodality manner [34].

As an alternative exogenously administered probes, multimodal approaches may be also based on reporter gene assays, in particular fusion proteins combining bioluminescence with thymidine kinase as PET probe [149]. Such dual optical-nuclear imaging approaches might yield improved quantification accuracy [150,151].

For example, fusion reporter gene assay have been used for non-invasive molecular imaging of protein-protein interactions *in vivo* with help of PET and *ex vivo* fluorescence microscopy [152]. Even a triple-fusion reporter gene has been proposed as an efficient tool for multimodality imaging combining fluorescence, bioluminescence and nuclear imaging techniques to investigate molecular processes [153–155].

## 4. Application in Pharmaceutical Research

**Figure 6.**
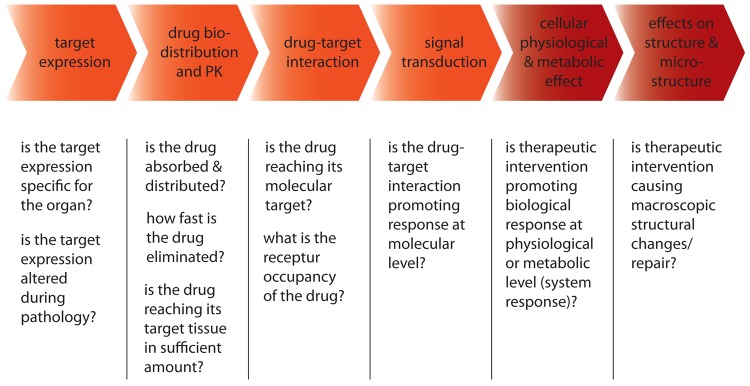
Multimodality imaging in drug discovery: Molecular imaging contributions are indicated in light red and relate to aspects addressing expression, PK and drug effects at a molecular level (receptor interaction and signaling). Physiological and structural readouts of drug efficacy are indicated in dark red.

The potential contribution of non-invasive imaging to the drug discovery and development process is illustrated in Figure 6. The process starts with the identification of a potential drug target, *i.e.*, molecular entity or process that is associated with the pathological condition and should be modified by the therapeutic intervention. Molecular drug targets include membrane receptors and channels, enzymes, proteins in signaling pathways, nuclear receptors, genes and the gene expression machinery. Target identification is based on clinical findings, e.g., altered expression in pathologic tissue, gene linkage studies, mechanistic consideration, etc. Validation of a target is of prime importance in a drug discovery program—a potential role for molecular imaging. There are numerous imaging studies illustrating the expression of molecular targets under pathological conditions. The next step in the cascade is the development of a screening assay suited for screening large compound libraries. After validation, compounds of interest will be further evaluated in animal models of human disease. Finally, the most important compounds will enter the clinical phase for early exploratory studies and eventually full clinical development.

### 4.1. Target Validation: Expression of Disease-Specific Molecular Targets

The conventional method to assess the presence of a molecular entity *in vivo* is the use of a target specific probe consisting of a targeting moiety linked to a reporter moiety. The targeting moiety typically consists of a low molecular weight drug analog, small peptide, antibody or antibody fragment, characterized by high affinity to their molecular target. The reporter moiety depends on the imaging modality used, for optical imaging it is typically a fluorescent dye or a quantum dot. It is important that the linking of the two molecular scaffolds does not affect the pharmacophor of the targeting molecule. This approach has been widely used to visualize the expression of receptors/antigens in tissue. Examples are the visualization of somatostatin receptors in neuroendocrine tumors in the mouse using a stabilized somatostatin analogue (octreotide) labeled with the fluorescent dye Cy5 [156], or of the expression of a fibronectin domain in activated endothelium that is associated with tumor neo-angiogenesis using a single chain antibody fragment labeled Cy7 [157]. Both molecular targets qualify as potential drug targets. Octrotide and related compounds are clinically used as anti-tumor agents, and inhibition of specific receptors expressed on activated endothelium may inhibit neo-angiogenesis. A disadvantage of this imaging strategy is that both target-bound and unbound probes will contribute to the signal. Correspondingly, contrast, or to more precise, the TBR is governed by the expression level of the molecular target and the pharmacokinetic properties of the probe molecule itself. If unbound probes clear slowly from the system, it may become difficult to achieve high TBR values. This has prompted the concept of activatable probes.

The fluorescence signal depends on the molecular environment of the fluorophore, which may affect the fluorescence wavelength of the fluorescence quantum yield. This has been exploited in the development of molecular beacons or self-quenched probes such as protease activatable probe [39]. In this probe design fluorescence is quenched unless a specific peptide motif is cleaved by the respective protease. For such probes, unprocessed circulating probe molecules will not contribute to the signal, which leads to a significant increase in the TBR. This probe concept has been used to study protease expression in tumors, which are among others associated with the degradation of the extracellular matrix and hence to the invasive nature of the tumor. Proof-of-concept was established in subcutaneously implanted human fibroscarcoma xenografts [39], and related probes have been used to study orthotopic gliomas in mice [1], mammary murine tumors in lung metastases in mice [69], or human mammary adenocarcinoma cells implanted in the mammary fat pad [44] to mention a few studies. Aside from their association to tumors, protease are expressed in inflammatory diseases such as atherosclerosis or rheumatoid and osteoarthritis. Optical imaging and FMT have been applied to monitor protease activity in genetically engineered mice that expressed a high burden of atherosclerotic lesions [76] and in collagen-induced models of rheumatoid arthritis [47].

### 4.2. Characterization of Disease Models/Disease Processes

Multimodal imaging has been widely used to characterize animal models of human disease based on structural, functional/physiological, metabolic, cellular and molecular readouts [158]. Concepts for targeting disease-specific molecular entities has been discussed in the previous section. Yet, the reader should be aware that not every specific molecular target presented by pathologically transformed tissue qualifies as drug target. For example, the occurrence of aggregated *β*-amyloid molecules in the brain parenchyma is a histopathological hallmark of Alzheimer's disease and therefore potential target for Alzheimer's diagnostic. Not surprisingly a large number of molecular imaging probes has been developed including some fluorescent molecules such as AOI987, an oxazine dye. Planar fluorescent imaging as well as FMT have been applied to monitor the deposition of *β*-amyloid plaques in murine models of Alzheimer's disease [85,86]. While not a therapeutic target per se, the method can be readily applied to stage the severity of the disease and to assess the efficacy of plaque modulating therapy. It is in principle translatable into the clinics, the clinical version of the assay is based on PET technology [159].

Visualization of the presence of a specific cell population and in particular their migration is of relevance for many applications in biomedical research. Inflammatory processes involve the orchestrated infiltration of various cell types such as lymphocytes, leukocytes and monocytes. Stable cell marking using bioluminescent and fluorescent reporter proteins is a commonly used approach to follow the migratory properties of immune competent cells. For example, a transgenic mouse model with a GFP reporter assay was established [160] and was further modified later on to study the response of the immune system after brain ischemia by imaging T lymphocytes [68]. Alternatively monocytes might be efficient labeled in situ using nanoparticulate moieties such as quantum dots. This approach has been applied to monitor the migration of monocytes to the sentinel lymphnodes [161]. Similar cell marking strategies have been applied to stably marking of tumor cells for monitoring the formation of brain liver and bone metastasis [49].

The function and processes involved in the immune response in different disease models can be investigated and quantified with FMT techniques. An interesting approach was chosen by Zaheer and coworkers [48] who constructed a NIR fluorescent bisphosphonate derivative that binds to hydroxyapatite which is used during bone formation and is produced by osteoblasts. Therefore they were able to study osteoblastic activity in skeletal developments in mice. Recently a fluorescence reporter probe was designed whose activation by cathepsin K was shown in osteoclast cells [162]. These examples showed the potential application for optical molecular imaging in fields where the research activity is strongly focussed on high resolution technologies such as CT or MRI. It was even possible to quantify the readout with help of FMT which was a novelty in the field.

### 4.3. Biomarkers to Assess Drug Efficacy

Biomarkers are objectively measurable quantities of prognostic quality that can predict how a pathology evolves or if there will be an effect due to a therapeutic intervention. For example, using AOI987 it has been shown that the plaque load in transgenic models of Alzheimer's disease increases as function of age of the mice [85], correspondingly the plaque load may be considered as a marker of disease severity. Similarly, one would expect a reduced plaque load in the case of a plaque modulating intervention. On the other hand a biomarker is not necessarily a surrogated endpoint. If we stay with our example, it is not warranted that a reduced plaque load will translate into an improved behavioral outcome, and if we translate into clinical situation, into improved cognitive performance. It is important to realize the difference between biomarkers and surrogates: Surrogates are validated measures that substitute for an endpoint (e.g., survival of a cancer patient), while a biomarker gives information whether the patient will respond to treatment. To what extend this predicts longterm outcome remains to be shown. Such biomarkers are highly attractive when studying chronic diseases.

Alternatively biomarkers may yield information on the validity of the pharmacological principle. As discussed in Section 4.1 high protease expression is associated with different diseases and inhibition of protease activity is therefore an attractive therapeutic concept, e.g., in cancer or inflammatory diseases. The protease imaging methods discussed may be used to evaluate such drugs. For example it has been shown that in the collagen-induced arthritis model fluorescence imaging using protease activatable probes accurately captures the effects of methotrexate treatment [47]. Similarly, administration of matrix metalloproteinase (MMP) inhibitor prinomastat reduced the signal elicited by protease activatable probes in HT1080 fibrosarcoma xenografts [38]. In both cases, the imaging experiment revealed information that the drugs reached their molecular targeted and inhibited the enzyme activity as expected. Wether this translates into an improved outcome, *i.e.*, inhibition of inflammatory processes and reduction of joint destruction in the arthritis model, or tumor shrinkage and eventually animal survival in the cancer study remains to be shown.

## 5. Potential and Limitation of Fluorescence Imaging

### 5.1. As Preclinical Research Tool

There is no doubt that the fast development of new reporter technologies had a significant impact on the role of fluorescence imaging in preclinical research. Optical reporter systems are in general stable and therefore suited for studying biological processes with time scales of days or weeks. On the other hand, the actual measurement process in live animals is rather fast, in particular when focusing on two-dimensional techniques, enabling the necessary high throughput for compound evaluation.

Luminescent signals can be modulated by molecular processes. This is a highly attractive property as probes can be designed in such a way that they generate a signal when the interaction with the molecular target takes place. Examples discussed are protease activatable probes which only yield a signal, when the protease specific peptide motif is cleaved [39]. This will result in a high TBR, as non-processed probe should not contribute to the fluorescence detected. Such probe designs are not conceivable for nuclear imaging approaches.

Advanced optical imaging techniques such as FMT provide quantitative information on local fluorophore concentration, which is critical e.g., when comparing the efficacy of different drugs. Optical imaging is a non-invasive research tool and does not cause radiation damage, which might be an issue with CT. With modern FMT systems imaging is no longer confined to superficial structures. Also sources located at distances of 10 to 20 mm from the surface can be accurately located and quantitatively assessed.

The principal technical limitation of FMT is the poor spatial resolution rendering the allocation of a fluorescent signal to a specific anatomical structure difficult. This is due to the limited number of source-detector pairs available for data reconstruction and the relatively crude models used for photon propagation in tissue, which is based on simplifications that most likely are not always appropriate. In addition, tissue optical parameters are not well defined. These issues have been addressed in part by combining high resolution structural imaging modalities with the optical readout either via sequential or simultaneous data acquisition. Considering actual anatomical information in the data reconstruction better confines the problem.

While it is straightforward to tune the excitation and emission wavelengths of organic dyes to fit the wavelength domain suitable for *in vivo* imaging, this is quite an issue when using fluorescent protein as reporters. For example GFP is characterized by high brightness (high quantum yield), but unfortunately in the wavelength range around 490 to 510 nm, in which absorption is an order of magnitude higher than in the NIR region. In addition, tissue autofluorescence is high at these wavelengths and will compromise TBR achievable. Autofluorescence of tissue caused by the excitation wavelength is always an issue in fluorescence imaging but can be strongly reduced by using further red shifted excitation wavelength [163]. Autofluorescence limits the capability of fluorescence imaging approaches to resolve deep seated structures as the penetration depth of green light is limited and the emitted light is strongly attenuated in tissue. Unspecific fluorescent signals can also originate from the animal's chow and can be eliminated by using a chlorophyll-free diet [164].

There have been significant efforts finding new proteins or genetically modify existing ones in order to shift the absorption and emission maxima to the red and NIR spectral domain with considerable success [31]. Yet the wavelength shifts achieved are not yet sufficient and the quantum yield of theses mutated proteins is significantly lower than that of GFP. Very recently a first report on an infrared fluorescent protein has been published [165].

A further factor impairing fluorescence imaging is skin pigmentation when using standard C57B/L6 mice. In areas of dark black pigmentation on the skin after fur removal, imaging may be severely compromised or virtually impossible due to enhanced light absorption. The use of nude mice could be a solution, but their use might be limited in many cases due to their compromised immune suppression. Also, most transgenic mouse lines exist on C57B/L6 or 129 background and are not available on a nude mouse background.

Today fluorescence imaging allowing whole body scanning in mice is a widely used methodology in experimental biomedical research. The method is sensitive, selective relatively cheap and allows for reasonable throughput. By carefully addressing the limiting factors, the method can be efficiently used to study molecular processes *in vivo*.

### 5.2. As Translational Research Tool

DOT imaging was first introduced as a clinical diagnostic tool for imaging the female breast. Breast tissue has relatively low attenuation and scattering coefficients and is therefore suitable for optical imaging. DOT was evaluated as screening modality for mammary carcinomas, which could be clearly visualized due to abnormal hypervascularity and increased hemoglobin absorbance. TBRs might be enhanced by administration of intravascular fluorescent dyes such as ICG [166]. Fluorescence enhanced imaging showed potential to replace the harmful and uncomfortable mammography as the gold standard. Recently an optical device to investigate prostate cancer was developed, which appeared promising to replace in the near future the painful clinical standard approach of biopsy [121], and DOT has been suggested as screening device for osteoarthritis by studying finger joints [99,100]. Yet all those studies are in an early exploratory phase. Substantial efforts are required to develop these methods into clinical diagnostic tools suited for screening purposes, with applications in breast cancer being the most advanced. Another clinical application of optical imaging is the assessment of brain function/brain oxygenation in neonates or even adults. Yet this approach has only limited connections to fluorescence optical imaging as it is based on spectroscopic analysis of hemoglobin and oxyhemoglobin signals, which are described elsewhere [167–169].

The use of fluorescence approaches in clinics is very limited due to the lack of clinically approved fluorescent probes. Therefore translation of preclinical optical imaging approaches might consist of translating a preclinical optical into a clinical nuclear imaging readout. An example might be the use of plaque-specific dyes in preclinical studies of mouse models of Alzheimer's disease and of PET probes such as Pittsburgh compound B (PIB) for the corresponding clinical application.

A strategic clinical application domain for which fluorescence imaging appears highly attractive is surgery. Realtime imaging might guide the surgeon in identifying the margins of malignant tissue, which will fluoresce during illumination with an appropriate wavelength to support dissection, when using tumor specific fluorescent markers. The high demands on speed and the fact that superficial information is required only renders planar imaging the method of choice.

The feasibility of imaging large human organs was demonstrated by Ntziachristos *et al.* [170] with simulation studies, from which the authors concluded that critical for accurate tissue identification is a high fluorescence TBR rather than limitation imposed by light propagation in tissue. While optical imaging is attractive for exposed single organs during surgery, it is not possible to image these organs in their natural location several centimeters below the surface and embedded within other tissue layers. Under these circumstances photon propagation becomes the limiting factor, which narrow down potential applications to low absorptive (e.g., adipose tissue, breast) and superficial (e.g., surgically exposed) tissue.

Thus, regarding clinical application optical imaging is covering potentially important niches for routinely clinical use.

## 6. Conclusions and Future Directions

Fluorescence imaging is a powerful tool for non-invasive investigation of biomedical systems at the molecular level. The targeted probes for optical imaging are easy to handle, widely available and do not require radiation protection measures as for probes involving radionuclides. In particular, FMT enabling to derive quantitative information form three-dimensional data sets is of interest for different biomedical research areas including pharmaceutical research.

FMT development is towards 360° whole body animal scanners which allows to freely select one or several regions of interest without need for moving the animal. Forward modeling will be further improved by incorporation of more realistic heterogeneous tissue models using either morphology standardized tissue layers that has to be adapted to a specific problem or alternatively the actual anatomical features of the object under study derived from a hybrid experiment. This requires combining FMT with structural imaging such as CT or MRI, a major direction of instrumental development. Even though CT provides sufficient contrast for identification of organ boundaries, MRI is superior in imaging soft tissue. Including anatomical constraints provided by these modalities in the reconstruction process will considerably improve the spatial accuracy of the reconstructed objects. Nevertheless, care has to be taken to not over constrain the outcome of the reconstruction, which would bias the interpretation of the results. Comprehensive understanding of biological processes is based on complementary information of structural functional and/or molecular nature.

Fully integrated solutions for simultaneous imaging are technically more demanding but offer substantial advantages such as inherent registration and the ability to monitor processes simultaneously. This translates into instrument design: FMT scanners are being developed as inserts to commercially available MRI scanners or mounted to the gantry of a CT scanner for full 360° space coverage. It is foreseeable that future high end FMT systems always designed as hybrids.

A key success factor for molecular imaging is the design of sensitive reporter systems. Developments are towards probes with better optical properties, *i.e.*, excitation and emission wavelength in the proper wavelength domain, higher sensitivity, *i.e.*, probes with better fluorescent quantum yield, smart probes to improve the target-to-background contrast. Similar to drug like molecules, target delivery might be an issue, in particular when targeting intramolecular targets.

Sensitive detection devices providing quantitative information on molecular processes in the intact organism are of high interest for basic biomedical research and in particular for therapy development. Molecular information is central for target validation, for pharmacokinetic studies, to demonstrate the pharmacological proof of principle (e.g., does a compound reach its target and inhibit the enzyme activity, Figure 6) and eventually can be used as a biomarker of compound efficacy. The translational potential of optical imaging is limited due to constraints regarding photon propagation in tissue, exceptions being breast imaging or intraoperative imaging. Nevertheless, the concept developed using fluorescent readouts in experimental animals might be translated into the clinics by replacing the fluorescent reporter by a radionuclide probe. This perspective renders optical imaging an attractive tool for pharmaceutical researchers both in view of experimental research and translation applications.

## References

[1] Ntziachristos V., Tung C.H., Bremer C., Weissleder R. (2002). Fluorescence molecular tomography resolves protease activity *in vivo*. Nat. Med..

[2] Ntziachristos V., Weissleder R. (2002). Charge-coupled-device based scanner for tomography of fluorescent near-infrared probes in turbid media. Med. Phys..

[3] Boas D.A., Brooks D.H., Miller E.L., DiMarzio C.A., Kilmer M., Gaudette R.J., Zhang Q. (2001). Imaging the Body with Diffuse Optical Tomography. IEEE Signal Process. Mag..

[4] Corlu A., Choe R., Durduran T., Rosen M.A., Schweiger M., Arridge S.R., Schnall M.D., Yodh A.G. (2007). Three-dimensional *in vivo* fluorescence diffuse optical tomography of breast cancer in humans. Opt. Express.

[5] Ntziachristos V., Yodh A.G., Schnall M., Chance B. (2000). Concurrent MRI and diffuse optical tomography of breast after indocyanine green enhancement. Proc. Natl. Acad. Sci. USA.

[6] Antcliff R.J., Stanford M.R., Chauhan D.S., Graham E.M., Spalton D.J., Shilling J.S., Ffytche T.J., Marshall J. (2000). Comparison between optical coherence tomography and fundus fluorescein angiography for the detection of cystoid macular edema in patients with uveitis. Ophthalmology.

[7] Ishimaru A. (1997). Wave Propagation and Scattering in Random Media.

[8] Arridge S.R. (1999). Optical tomography in medical imaging. Inverse Probl..

[9] Yoo K.M., Liu F., Alfano R.R. (1990). When does the diffusion approximation fail to describe photon transport in random media?. Phys. Rev. Lett..

[10] Li X., Chance B., Yodh A.G. (1998). Fluorescent heterogeneities in turbid media: limits for detection, charcterization, and comparison with absorption. Appl. Opt..

[11] O'Leary M., Boas D., Li X., Chance B., Yodh A. (1996). Fluorescence lifetime imaging in turbid media. Opt. Lett..

[12] Li X.D., O'Leary M.A., Boas D.A., Chance B., Yodh A.G. (1996). Fluorescent diffuse photon density waves in homogeneous and heterogeneous turbid media: Analytic solutions and applications. Appl. Opt..

[13] Kienle A., Patterson M.S. (1997). Improved solutions of the steady-state and the time-resolved diffusion equation for reflectance from a semi-infinite turbid medium. J. Opt. Soc. Am. A.

[14] Patterson M.S., Chance B., Wilson B.C. (1989). Time resolved reflectance and transmittance for the noninvasive measurement of tissue optical properties. Appl. Opt..

[15] van der Mark M.B., van Albada M.P., Lagendijk A. (1988). Light scattering in strongly scattering media: multiple scattering and weak localization. Phys. Rev. B.

[16] Ripoll J., Ntziachristos V., Culver J.P., Pattanayak D.N., Yodh A.G., Nieto-Vesperinas M. (2001). Recovery of optical parameters in multiple-layered diffusive media: theory and experiments. J. Opt. Soc. Am. A.

[17] Martelli F., Sassaroli A., Yamada Y., Zaccanti G. (2002). Analytical approximate solution of the time domain diffusion equation in layered slabs. J. Opt. Soc. Am. A.

[18] Kienle A. (2005). Light diffusion through a turbid parallelepiped. J. Opt. Soc. Am. A.

[19] Walker S.A., Boas D.A., Gratton E. (1998). Photon density waves scattered from cylindrical inhomogeneities: theory and experiments. Appl. Opt..

[20] Liemert A., Kienle A. (2010). Light diffusion in a turbid cylinder. II. Layered case. Opt. Express.

[21] Boas D.A., O'Leary M.A., Chance B., Yodh A.G. (1994). Scattering of diffuse density waves by spherical inhomogeneities within turbid media: Analytic solution and applications. Proc. Natl. Acad. Sci. USA.

[22] Arridge S.R., Cope M., Delpy D.T. (1992). The theoretical basis for the determination of optical pathlengths in tissue: temporal and frequency analysis. Phys. Med. Biol..

[23] Ripoll J., Nieto-Vesperinas M. (1999). Scattering integral equation for diffusive waves: detection of objects buried in diffusive media in the presence of rough interfaces. J. Opt. Soc. Am. A.

[24] Gibson A.P., Hebden J.C., Arridge S.R. (2005). Recent advances in diffuse optical imaging. Phys. Med. Biol..

[25] Ntziachristos V., Weissleder R. (2001). Experimental three-dimensional fluorescence reconstruction of diffuse media by use of a normalized Born approximation. Opt. Lett..

[26] Soubret A., Ripoll J., Ntziachristos V. (2005). Accuracy of fluorescent tomography in the presence of heterogeneities: Study of the normalized Born ratio. IEEE Trans. Med. Imaging.

[27] Roy R., Godavarty A., Sevick-Muraca E.M. (2003). Fluorescence-enhanced optical tomography using referenced measurements of heterogeneous media. IEEE Trans. Med. Imaging.

[28] Kak A.C., Slaney M. (2001). Principles of Computerized Tomographic Imaging.

[29] Gaudette R.J., Brooks D.H., DiMarzi C.A., Kilmer M.E., Miller E.L., Boas D.A. (2000). A comparison study of linear reconstruction techniques for diffuse optical tomographic imaging of absorption coefficient. Phys. Med. Biol..

[30] Arridge S.R., Schotland J.C. (2009). Optical tomography: forward and inverse problems. Inverse Probl..

[31] Shaner N.C., Steinbach P.A., Tsien R.Y. (2005). A guide to choosing fluorescent proteins. Nat. Methods.

[32] Tsien R.Y. (2005). Building and breeding molecules to spy on cells and tumors. FEBS Lett..

[33] Chatterjeea D.K., Rufaihaha A.J., Zhanga Y. (2008). Upconversion fluorescence imaging of cells and small animals using lanthanide doped nanocrystals. Biomaterials.

[34] Zhou J., sun Y., Du X., Xiong L., Hu H., Liuzzi F. (2010). Dual-modality *in vivo* imaging using rare-earth nanocrystals with near-infrared to near-infrared (NIR-to-NIR) upconversion luminescence and magnetic resonance properties. Biomaterials.

[35] Nyk M., Kumar R., Ohulchanskyy T.Y., Bergey E.J., Prasad P.N. (2008). High contrast *in vitro* and *in vivo* photoluminescence bioimaging using near infrared to near infrared up-conversion in Tm^3+^ and Yb^3+^ doped fluoride nanophosphors. Nano Lett..

[36] Tian Y., Cao W.H., Luo X.X., Fu Y. (2007). Preparation and luminescence property of Gd_2_O_2_S:Tb X-ray nano-phosphors using the complex precipitation method. J. Alloys Compounds.

[37] Wang H., Wang R., Sun X., Yan R., Li Y. (2005). Synthesis of red-luminescent Eu3+ doped lanthanides compounds hollow spheres. Mater. Res. Bull..

[38] Bremer C., Tung C.H., Weissleder R. (2001). *In vivo* molecular target assessment of matrix metalloproteinase inhibition. Nat. Med..

[39] Tung C.H., Mahmood U., Bredow S., Weissleder R. (2000). *In vivo* imaging of proteolytic enzyme activity using a novel molecular reporter. Cancer Res..

[40] Kobayashi H., Ogawa M., Alford R., Choyke P.L., Urano Y. (2010). New strategies for fluorescent probe design in medical diagnostic imaging. Chem. Rev..

[41] Han J., Burgess K. (2010). Fluorescent indicators for intracellular pH. Chem. Rev..

[42] Bünzli J.C. (2010). Lanthanide luminescence for biomedical analyses and imaging. Chem. Rev..

[43] Sevick-Muraca E.M., Houston J.P., Gurfinkel M. (2002). Fluorescence-enhanced, near infrared diagnostic imaging with contrast agents. Curr. Opin. Chem. Biol..

[44] Mahmood U., Tung C.H., Bogdanov A., Weissleder R. (1999). Near-infrared optical imaging of protease activity for tumor detection. Radiology.

[45] Ke S., Wen X., Gurfinkel M., Charnsangavej C., Wallace S., Sevick-Muraca E.M., Li C. (2003). Near-infrared optical imaging of epidermal growth factor receptor in breast cancer xenografts. Cancer Res..

[46] Weissleder R., Tung C.H., Mahmood U., Bogdanov A. (1999). *In vivo* imaging of tumors with protease-activated near-infrared fluorescent probes. Nat. Biotechnol..

[47] Wunder A., Tung C.H., Müller-Ladner U., Weissleder R., Mahmood U. (2004). *In vivo* imaging of protease activity in arthritis: A novel approach for monitoring treatment response. Arthritis Rheum..

[48] Zaheer A., Lenkinski R.E., Mahmood A., Jones A.G., Cantley L.C., Frangioni J.V. (2001). *In vivo* near-infrared fluorescence imaging of osteoblastic activity. Nat. Biotechnol..

[49] Yang M., Baranov E., Jiang P., Sun F.X., Li X.M., Hasegawa S., Bouvet M., Al-Tuwaijri M., Chishima T., Shimada H., Moossa A.R., Penman S., Hoffman R.M. (2000). Whole-body optical imaging of green fluorescent protein-expressing tumors and metastases. Proc. Natl. Acad. Sci. USA.

[50] Ntziachristos V. (2006). Fluorescence molecular imaging. Annu. Rev. Biomed. Eng..

[51] Cutler M. (1929). Transillumination as an aid in the diagnosis of breast lesions. Surg. Gynecol. Obstet..

[52] Ntziachristos V., Turner G., Dunham J., Windsor S., Soubret A., Ripoll J., Shih H.A. (2005). Planar fluorescence imaging using normalized data. J. Biomed. Opt..

[53] Farkas D.L., Du C., Fisher G.W., Lau C., Niu W., Wachman E.S., Levenson R.M. (1998). Non-invasive image acquisition and advanced processing in optical and bioimaging. Comput. Med. Imaging Graph..

[54] Gao X., Cui Y., Levenson R.M., Chung L.W.K., Nie S. (2004). *In vivo* cancer targeting and imaging with semiconductor quantum dots. Nat. Biotechnol..

[55] Ntziachristos V., Ripoll J., Wang L.V., Weissleder R. (2005). Looking and listening to light: the evolution of whole-body photonic imaging. Nat. Biotechnol..

[56] Hielscher A.H. (2005). Optical tomographic imaging of small animals. Curr. Opin. Biotechnol..

[57] Leblond F., Davis S.C., Valdés P.A., Pogue B.W. (2010). Pre-clinical whole-body fluorescence imaging: Review of instruments, methods and applications. J. Photochem. Photobiol. B: Biol..

[58] Turner G.M., Zacharakis G., Soubret A., Ripoll J., Ntziachristos V. (2005). Complete-angle diffuse optical tomography by use of early photons. Opt. Lett..

[59] Niedre M.J., de Kleine R.H., Aikawa E., Kirsch D.G., Weissleder R., Ntziachristos V. (2008). Early photon tomography allows fluorescence detection of lung carcinomas and disease progression in mice *in vivo*. Proc. Natl. Acad. Sci. USA.

[60] Ntziachristos V., Ma X., Chance B. (1998). Time-correlated single photon counting imager for simultaneous magnetic resonance and near-infrared mammography. Rev. Sci. Instrum..

[61] Culver J.P., Ntziachristos V., Holboke M.J., Yodh A.G. (2001). Optimization of optode arrangements for diffuse optical tomography: A singular-value analysis. Opt. Lett..

[62] Culver J.P., Choe R., Holboke M.J., Zubkov L., Durduran T., Slemp A., Ntziachristos V., Chance B., Yodh A.G. (2003). Three-dimensional diffuse optical tomography in the parallel plane transmission geometry: Evaluation of a hybrid frequency domain/continuous wave clinical system for breast imaging. Med. Phys..

[63] Graves E.E., Ripoll J., Weissleder R., Ntziachristos V. (2003). A submilimeter resolution fluorescence molecular imaging system for small animal imaging. Med. Phys..

[64] Zacharakis G., Ripoll J., Weissleder R., Ntziachristos V. (2005). Fluorescent protein tomography scanner for small animal imaging. IEEE Trans. Med. Imaging.

[65] Schulz R.B., Ripoll J., Ntziachristos V. (2004). Experimental fluorescence tomography of tissues with noncontact measurements. IEEE Trans. Med. Imaging.

[66] Ripoll J., Schulz R.B., Ntziachristos V. (2003). Free-space propagation of diffuse light: Theory and experiments. Phys. Rev. Lett..

[67] Schulz R.B., Ripoll J., Ntziachristos V. (2003). Noncontact optical tomography of turbid media. Opt. Lett..

[68] Martin A., Aguirre J., Sarasa-Renedo A., Tsoukatou D., Garofalakis A., Meyer H., Ripoll J., Planas A.M. (2008). Imaging changes in lymphoid organs *in vivo* after brain ischemia with three-dimensional fluorescence molecular tomography in transgenic mice expressing green fluorescent protein in T lymphocytes. Mol. Imaging.

[69] Koenig A., Hervé L., Josserand V., Berger M., Boutet J., Silva A.D., Dinten J.M., Peltié P. (2008). *In vivo* mice lung tumor follow-up with fluorescence diffuse optical tomography. J. Biomed. Opt..

[70] Meyer H., Garofalakis A., Zacharakis G., Psycharakis S., Mamalaki C., Kioussis D., Economou E.N., Ntziachristos V., Ripoll J. (2007). Non-contact optical imaging in mice with full angular coverage and automatic surface extraction. Appl. Opt..

[71] Deliolanis N., Lasser T., Hyde D., Soubret A., Ripoll J., Ntziachristos V. (2007). Free-space fluorescence molecular tomography utilizing 360° geometry projections. Opt. Lett..

[72] Lasser T., Soubret A., Ripoll J., Ntziachristos V. (2008). Surface reconstruction for free-space 360*^circ^* fluorescence molecular tomography and the effects of animal motion. IEEE Trans. Med. Imaging.

[73] Li C., Mitchell G.S., Dutta J., Ahn S., Leahy R.M., Cherry S.R. (2099). A three-dimensional multispectral fluorescence optical tomography imaging system for small animals based on a conical mirror design. Opt. Express.

[74] Siegel A.M., Mandeville J.B., Boas D.A. (2003). Temporal comparison of functional brain imaging with optical tomography and fMRI during rat forepaw stimulation. Phys. Med. Biol..

[75] McCann C.M., Waterman P., Figueiredo J.L., Aikawa E., Weissleder R., Chen J.W. (2009). Combined magnetic resonance and fluorescence imaging of the living mouse brain reveals glioma response to chemotherapy. Neuroimage.

[76] Nahrendorf M., Waterman P., Thurber G., Groves K., Rajopadhye M., Panizzi P., Marinelli B., Aikawa E., Pittet M.J., Swirski F.K., Weissleder R. (2009). Hybrid *in vivo* FMT-CT imaging of protease activity in atherosclerosis with customized nanosensors. Artheroscler. Thromb. Vasc. Biol..

[77] Allard M., Côté D., Davidson L., Dazai J., Henkelman R.M. (2007). Combined magnetic resonance and bioluminescence imaging of live mice. J. Biomed. Opt..

[78] Celentano L., Laccetti P., Liuzzi R., Mettivier G., Montesi M.C., Autiero M., Riccio P., Roberti G., Russo P., Salvatore M. (2003). Preliminary tests of a prototype system for optical and radionuclide imaging in small animals. IEEE Trans. Nucl. Sci..

[79] Autiero M., Celentano L., Cozzolino R., Laccetti P., Marotta M., Mettivier G., Montesi M.C., Riccio P., Roberti G., Russo P. (2005). Experimental study on *in vivo* optical and radionuclide imaging in small animals. IEEE Trans. Nucl. Sci..

[80] Joshi A., Rasmussen J.C., Kwon S., Wareing T.A., McGhee J., Sevick-Muraca E.M. Multi-modality CT-PET-NIR fluorescence tomography.

[81] Deroose C.M., De A., Loening A.M., Chow P.L., Ray P., amd S.S., Gambhir A.F.C. (2007). Multimodality imaging of tumor xenografts and metastases in mice with combined small-animal PET, small-animal CT, and bioluminescence imaging. J. Nucl. Med..

[82] Ale A., Schulz R.B., Sarantopoulos A., Ntziachristos V. (2010). Imaging performance of a hybrid X-ray computed tomography-fluorescence tomography system using priors. Med. Phys..

[83] Zacharakis G., Kambara H., Shih H., Ripoll J., Grimm J., Saeki Y., Weissleder R., Ntziachristos V. (2005). Volumetric tomography of fluorescent proteins through small animals *in vivo*. Proc. Natl. Acad. Sci. USA.

[84] Licha K., Lin C.P. (2009). Dual-modality molecular imaging for small animals using fluorescence and X-ray computed tomography, Molecular Imaging II. Proc. Soc. Photo-Opt. Instrum. Eng..

[85] Hintersteiner M., Enz A., Frey P., Jaton A.L., Kinzy W., Kneuer R., Neumann U., Rudin M., Staufenbiel M., Stoeckli M., Wiederhold K.H., Gremlich H.U. (2005). *In vivo* detection of amyloid-*β* deposits by near-infrared imaging using an oxazine-derivative probe. Nat. Biotechnol..

[86] Hyde D., de Kleine R., MacLaurin S.A., Miller E., Brooks D.H., Krucker T., Ntziachristos V. (2009). Hybrid FMT-CT imaging of amyloid-*β* plaques in a murine Alzheimer's disease model. Neuroimage.

[87] Kepshire D., Mincu N., Hutchins M., Gruber J., Dehghani H., Hypnarowski J., Leblond F., Khayat M., Pogue B.W. (2009). A microcomputed tomography guided fluorescence tomography system for small animal molecular imaging. Rev. Sci. Instrum..

[88] Barber W.C., Lin Y., Nalcioglu O., Iwanczyk J.S., Hartsough N.E., Gulsen G. (2010). Combined fluorescence and X-ray tomography for quantitative *in vivo* detection of fluorophore. Tech. Canc. Res. Treat..

[89] Lin Y., Barber W.C., Iwanczyk J.S., Roeck W., Nalcioglu O., Gulsen G. (2010). Quantiative fluorescence tomography using a combined tri-modality FT/DOT/XCT system. Opt. Express.

[90] Lin Y., Barber W.C., Iwanczyk J.S., Roeck W.W., Nalcioglu O., Gulsen G. (2010). Quantiative fluorescence tomography using a trimodality system: *in vivo* validation. J. Biomed. Opt. Lett..

[91] Schulz R.B., Ale A., Sarantopoulos A., Freyer M., Soehngen E., Zientkowska M., Ntziachristos V. (2010). Hybrid system for simultaneous fluorescence and X-ray computed tomography. IEEE Trans. Med. Imaging.

[92] Licha K., Lin C.P. (2009). Hybrid fluorescence tomography/X-ray tomography improves reconstruction quality, Molecular Imaging II. Proc. Soc. Photo-Opt. Instrum. Eng..

[93] Yang X., Gong H., Quan G., Deng Y., Luo Q. (2010). Combined system of fluorescence diffuse optical tomography and microcomputed tomography for small animal imaging. Rev. Sci. Instrum..

[94] da Silva A., Leabad M., Bordy T., Dinten J.M., Peltié P., Rizo P. (2007). Design of a small animal multimodality tomographer for x-ray and optical coupling: Theory and experiments. Nucl. Instr. Meth. Phys. Res. A.

[95] Silva A.D., Leabad M., Driol C., Bordy T., Debourdeau M., Dinten J.M., Peltié P., Rizo P. (2009). Optical calibration protocol for an x-ray and optical multimodality tomography system dedicated to small-animal examination. Appl. Opt..

[96] Liu J., Wang Y., Qu X., Li X., Ma X., Han R., Hu Z., Chen X., Sun D., Zhang R., Chen D., Chen D., Chen X., Liang J., Cao F., Tian J. (2010). *In vivo* quantitative bioluminescence tomography using heterogeneous and homogeneous mouse models. Opt. Express.

[97] Zhang Q., Brukillacchio T.J., Li A., Stott J.J., Chaves T., Hillman E., Wu T., Chorloton M., Rafferty E., Moore R.H., Kopans D.B., Boas D.A. (2005). Coregistered tomographic x-ray and optical breast imaging: initial results. J. Biomed. Opt..

[98] Li A., Miller E.L., Kilmer M.E., Brukilacchio T.J., Chaves T., Stott J., Zhang Q., Wu T., Chorlton M., Moore R.H., Kopans D.B., Boas D.A. (2003). Tomographic optical breast imaging guided by three-dimensional mammography. Appl. Opt..

[99] Yuan Z., Zhang Q., Sobel E., Jiang H. (2007). Three-dimensional diffuse optical tomography of osteoarthritis: initial results in the finger joints. J. Biomed. Opt..

[100] Yuan Z., Zhang Q., Sobel E.S., Jiang H. (2008). Tomographic X-ray-guided three-dimensional diffuse optical tomography of osteoarthritis in the finger joints. J. Biomed. Opt..

[101] Carpenter C.M., Sun C., Pratx G., Rao R., Xing L. (2010). Hybrid X-ray/optical luminescence imaging: Characterization of experimental conditions. Med. Phys..

[102] Pratx G., Carpenter C.M., Sun C., Xing L. (2010). X-ray luminescence computed tomography via selective excitation: A feasibility study. IEEE Trans. Med. Imaging.

[103] Davis S.C., Samkoe K.S., O'Hara J.A., Gibbs-Strauss S.L., Paulsen K.D., Pogue B.W. (2010). Comparing implementations of magnetic-resonance-guided fluorescence molecular tomography for diagnostic classification of brain tumors. J. Biomed. Opt..

[104] Tromberg B.J., Pogue B.W., Paulsen K.D., Yodh A.G., Boas D.A., Cerussi A.E. (2008). Assessing the future of diffuse optical imaging technologies for breast cancer managment. Med. Phys..

[105] Azar F.S., Intes X. (2009). *MRI*-guided fluorescence tomography of the breast: A phantom study, Multimodel Biomedical Imaging IV. Proc. Soc. Photo-Opt. Instrum. Eng..

[106] Davis S.C., Dehghani H., Wang J., Jiang S., Pogue B.W., Paulsen K.D. (2007). Image-guided diffuse optical fluorescence tomography implemented with Laplacian-type regularization. Opt. Express.

[107] Davis S.C., Pogue B.W., Springett R., Leussler C., Mazurkewitz P., Tuttle S.B., Gibbs-Strauss S.L., Jiang S.S., Dehghani H., Paulsen K.D. (2008). Magnetic resonance-coupled tomography scanner for molecular imaging of tissue. Rev. Sci. Instrum..

[108] Azar F.S., Intes X. (2008). *MRI*-coupled spectrally-resolved fluorescence tomography for *in vivo* imaging. Proc. Soc. Photo-Opt. Instrum. Eng..

[109] Davis S.C., Samkoe K.S., O'Hara J.A., Gibbs-Strauss S.L., Payne H.L., Hoopes J., Paulsen K.D., Pogue B.W. (2010). MRI-coupled fluorescence tomography quantifies EGFR activity in brain tumor. Acad. Radiol..

[110] Xu H., Springet R., Dehghani H., Pogue B.W., Paulsen K.D., Dunn J.F. (2005). Magentic-resonance-imaging-coupled broadband near-infrared tomography system for small animal brain studies. Appl. Opt..

[111] Gulsen G., Birgul O., Unlu M.B., Shafiiha R., Nalcioglu O. (2006). Combined diffuse optical tomography (DOT) and MRI system for cancer imaging in small animals. Tech. Canc. Res. Treat..

[112] Zhang X., Toronov V.Y., Webb A.G. (2005). Simultaneous integrated diffuse optical tomography and functional magnetic resonance imaging of the human brain. Opt. Express.

[113] Huppert T.J., Diamond S.G., Boas D.A. (2008). Direct estimation of evoked hemoglobin changes by multimodality fusion imaging. J. Biomed. Opt..

[114] Ntziachristos V. (2010). Going deeper than microscopy: optical imaging frontier in biology. Nat. Methods.

[115] Razansky D., Distel M., Vinegoni C., Perrimon N., Köster R.W., Ntziachristos V. (2009). Multispectral opto-acoustic tomography of deep-seated fluorescent proteins *in vivo*. Nat. Photon..

[116] Boutet J., Hervé L., Debourdeau M., Guyon L., Peltié P., Dinten J.M., Saroul L., Duboeuf F., Vray D. (2009). Bimodal ultrasound and fluorescence approach for prostate cancer diagnosis. J. Biomed. Opt..

[117] Holboke M.J., Tromberg B.J., Li X., Shah N., Fishkin J., Kidney D., Butler J., Chance B., Yodh A.G. (2000). Three-dimensional diffuse optical mammography with ultrasound localization in human subject. J. Biomed. Opt..

[118] Zhu Q., Durduran T., Ntziachristos V., Holboke M., Yodh A.G. (1999). Imager that combines near-infrared diffuse light and ultrasound. Opt. Lett..

[119] Zhu Q., Chen N., Kurtzman S.H. (2003). Imaging tumor angiogenesis by use of combined near-infrared diffuse light and ultrasound. Opt. Lett..

[120] Xu G., Piao D., Musgrove C.H., Bunting C.F., Dehghani H. (2008). Trans-rectal ultrasound-coupled near-infrared optical tomography of prostate Part I: Simulation. Opt. Express.

[121] Jiang Z., Piao D., Xu G., Ritchey J.W., Holoyoak G.R., Bartels K.E., Bunting C.F., Slobodov G., Krasinki J.S. (2008). Trans-rectal ultrasound-coupled near-infrared optical tomography of the prostate Part II: Experimental demonstration. Opt. Express.

[122] Jiang Z., Holyoak G.R., Bartels K.E., Ritchey J.W., Xu G., Bunting C.R., Slobodov G., Piao D. (2009). *In vivo* trans-rectal ultrasound-coupled optical tomography of a transmissible venereal tumor model in the canine pelvic canal. J. Biomed. Opt..

[123] Culver J., Akers W., Achilefu S. (2008). Multimodality molecular imaging with combined optical and SPECT/PET modalities. J. Nucl. Med..

[124] Prout D.L., Silverman R.W., Chatziioannou A. (2004). Detector concept for OPET, a combined PET and optical imaging system. IEEE Trans. Nucl. Sci..

[125] Jung J.H., Choi Y., Hong K.J., Min B.J., Choe J.Y.C.Y.S., Lee K.H., Kim B.T. (2009). Development of a dual modality imaging system: a combined gamma camera and optical imager. Phys. Med. Biol..

[126] Prout D.L., Silverman R.W., Chatziioannou A. (2005). Readout of the optical PET (OPET) detector. IEEE Trans. Nucl. Sci..

[127] Douraghy A., Rannou F.R., Silverman R.W., Chatziioannou A.F. (2008). FPGA electronics for OPET: A dual-modality optical and positron emission tomograph. IEEE Trans. Nucl. Sci..

[128] Vu N.T., Silverman R.W., Chatziioannou A.F. (2006). Preliminary performance of optical PET (OPET) detectors for the detection of visible light photons. Nucl. Instrum. Meth Phys. Res. A.

[129] Alexandrakis G., Rannou F.R., Chatziioannou A.F. (2005). Tomographic bioluminescence imaging by use of a combined optical-PET (OPET) system: a computer simulation feasibility study. Phys. Med. Biol..

[130] Alexandrakis G., Rannou F.R., Chatziioannou A.F. (2006). Effect of optical properties estimation accuracy on tomographic bioluminescence imaging: Simulation of a combined optical-PET (OPET) system. Phys. Med. Biol..

[131] Rannou F.R., Kohli V., Prout D.L., Chatziioannou A.F. (2004). Investigation of OPET performance using GATE a geant4-based simulation software. IEEE Trans. Nucl. Sci..

[132] Douraghy A., Prout D.L., Silverman R.W., Chatziioannou A.F. (2006). Evaluation of scintillator afterglow for use in a combined optical and PET imaging tomograph. Nucl. Instrum. Meth. Phys. Res. A.

[133] Peter J., Unholtz D., Schulz R.B., Semmler W. (2007). Development and initial results of a tomographic dual-modality positron/optical small animal imager. IEEE Trans. Nucl. Sci..

[134] Nahrendorf M., Keliher E., Marinelli B., Waterman P., Feruglio P.F., Fexon L., Pivovarov M., Swirski F.K., Pittet M.J., Vinegoni C., Weissleder R. (2010). Hybrid PET-optical imaging using targeted probes. Proc. Natl. Acad. Sci. USA.

[135] Garofalakis A., Dubois A., Kühnast B., Dupont D.M., Janssens I., Mackiewicz N., Dollé F., Tavitian B., Ducongé F. (2010). *In vivo* validation of free-space fluorescence tomography using nuclear imaging. Opt. Lett..

[136] Robertson R., Germanos M.S., Li C., Mitchell G.S., Cherry S.R., Silva M.D. (2009). Optical imaging of Cerenkov light generation from positron-emitting radiotracers. Phys. Med. Biol..

[137] Liu H., Ren G., Miao Z., Zhang X., Tang X., Han P., Gambhir S.S., Cheng Z. (2010). Molecular optical imaging with radioactive probes. PLoS One.

[138] Hyde D., Schulz R., Brooks D., Miller E., Ntziachristos V. (2009). Performance dependence of hybrid X-ray computed tomography/fluorescence molecular tomography on the optical forward problem. J. Opt. Soc. Am. A.

[139] Tan Y., Jiang H. (2008). Diffuse optical tomography guided quantitative fluorescence molecular tomography. Appl. Opt..

[140] Hervé L., Koenig A., Silva A.D., Berger M., Boutet J., Dinten J.M., Peltié P., Rizo P. (2007). Noncontact fluorescence diffuse optical tomography of heterogeneous media. Appl. Opt..

[141] Milstein A.B., Oh S., Webb K.J., Bouman C.A., Zhang Q., Boas D.A., Millane R.P. (2003). Fluorescence optical diffusion tomography. Appl. Opt..

[142] Lin Y., Yan H., Nalcioglu O., Gulsen G. (2009). Quantitative fluorescence tomography with functional and structural *a priori* information. Appl. Opt..

[143] Lin Y., Gao H., Nalcioglu O., Gulsen G. (2007). Fluorescence diffuse optical tomography with functional and anatomical *a priori* information: feasibility study. Phys. Med. Biol..

[144] Li D., Wang W., Wu Q., Ke S., Houston J., Sevick-Muraca E., Dong L., Chow D., Charnsangavej C., Gelovani J.G. (2006). Dual optical and nuclear imaging in human melanoma xenografts using a single targeted imaging probe. Nucl. Med. Biol..

[145] Edwards W.B., Akers W.J., Ye Y., Cheney P.P., Bloch S., Xu B., Laforest R., Achilefu S. (2009). Multimodal imaging of integrin receptor-positive tumors by bioluminescence, fluorescence, gamma scintigraphy and SPECT methods using a cyclic RGD peptide labeled with a near infrared fluorescent dye and a radionuclide. Mol. Imaging.

[146] Lee H., Akers W.J., Cheney P.P., Edwards W.B., Liang K., Culver J.P., Achilefu S. (2009). Complementary optical and nuclear imaging of caspase-3 activity using combined activatable and radio-labeled multimodality molecular probe. J. Biomed. Opt. Lett..

[147] Josephson L., Kircher M.F., Mahmood U., Tang Y., Weissleder R. (2002). Near-infrared fluorescencent nanoparticles as combined MR/optical imaging probes. Bioconjugate Chem..

[148] Shan L., Wang S., Sridhar R., Bhujwalla Z.M., Wang P.C. (2007). Dual probe with fluorescent and magnetic properties for imaging solid tumor xenografts. Mol. Imaging.

[149] Ray P., Wu A.M., Gambhir S.S. (2003). Optical bioluminescence and positron emission tomography imaging of a novel fusion reporter gene in tumor xenografts of living mice. Cancer Res..

[150] Cai W., Chen K., Gambhir S.S., Chen X. (2007). Dual-function probe for PET and near-infrared fluorescence imaging of tumor vasculature. J. Nucl. Med..

[151] Ducongé F., Pons T., Pestourie C., Hérin L., Thézé B., Gombert K., Mahler B., Hinnen F., Dollé B.K.F., Dubertret B., Tavitian B. (2008). Fluorine-18-labeled phospholipid quantum dot micelles for *in vivo* multimodal imaging from whole body to cellular scales. Bioconjugate Chem..

[152] Luker G.D., Sharma V., Pica C.M., Dahlheimer J.L., Li W., Ochesky J., Ryan C.E., Piwnica-Worms H., Piwnica-Worms D. (2002). Noninvasive imaging of protein-protein interactions in living animals. Proc. Natl. Acad. Sci. USA.

[153] Ponomarev V., Doubrovin M., Sereganova I., Vider J., Shavrin A., Beresten T., Ivanova A., Ageyeva L., Tourkova V., Balatoni J., Bornmann W., Blasberg R., Tjuvajev R.G. (2004). A novel triple-modality reporter gene for whole-body fluorescent bioluminescent and nuclear noninvasive imaging. Eur. J. Nucl. Med. Mol. Imaging.

[154] Ray P., De A., Min J.J., Tsien R.Y., Gambhir S.S. (2004). Imaging tri-fusion multimodality reporter gene expression in living subjects. Cancer.

[155] Blasberg R.G. (2003). *In vivo* molecular-genetic imaging: multi-modality nuclear and optical combinations. Nucl. Med. Biol..

[156] Becker A., Hassenius C., Licha K., Ebert B., Sukowski U., Semmler W., Wiedmann B., Grötzinger C. (2001). Receptor-targeted optical imaging of tumors with near-infrared fluorescent ligands. Nat. Biotechnol..

[157] Birchler M., Neri G., Tarli L., Halin C., Viti F., Neri D. (1999). Infrared photodetection for the *in vivo* localisation of phage-derived antibodies directed against angiogenic markers. J. Immunol. Methods.

[158] Rudin M., Rausch M., Stoeckli M. (2005). Molecular imaging in drug discovery and development: potential and limitations of nonnuclear methods. Mol. Imaging Biol..

[159] Klunk W.E., Engler H., Nordberg A., Wang Y., Blomqvist G., Holt D.P., Bergström M., Savitcheva I., Huang G.F., Estrada S., Ausén B., Debnath M.L., Barletta J., Price J.C., Sandell J., Lopresti B.J., Wall A., Koivisto P., Antoni G., Mathis C.A., Langström B. (2004). Imaging brain amyloid in Alzheimer's disease with Pittsburgh Compound-B. Ann. Neurol..

[160] Garofalakis A., Zacharakis G., Meyer H., Economou E.N., Mamalaki C., Papamatheakis J., Kioussis D., Ntziachristos V., Ripoll J. (2007). Three-dimensional *in vivo* imaging of green fluorescent protein-expressing T cells in mice with noncontact fluorescence molecular tomography. Mol. Imaging.

[161] Kim S., Lim Y.T., Soltesz E.G., Grand A.M.D., Lee J., Nakayama A., Parker J.A., Mihaljevic T., Laurence R.G., Dor D.M., Cohn L.H., Bawendi M.G., Frangioni J.V. (2004). Near-infrared fluorescent type II quantum dots for sentinel lymph node mapping. Nat. Biotechnol..

[162] Kozloff K.M., Quinti L., Patntirapong S., Hauschka P.V., Tung C.H., Weissleder R., Mahmood U. (2009). Non-invasive optical detection of cathepsin K-mediated fluorescence reveals osteoclast activity *in vitro* and *in vivo*. Bone.

[163] Weissleder R., Ntziachristos V. (2003). Shedding light onto live molecular targets. Nat. Med..

[164] Bouchard M.B., MacLaurin S.A., Dwyer P.J., Mansfield J., Levenson R., Krucker T. (2007). Technical considerations in longitudinal multispectral small animal molecular imaging. J. Biomed. Opt..

[165] Shu X., Wang L., Colip L., Kallio K., Remington S.J. (2009). Unique interactions between the chromophore and glutamate 16 lead to far-red emission in a red fluorescence protein. Protein Sci..

[166] Intes X., Ripoll J., Chen Y., Nioka S., Yodh A.G., Chance B. (2003). *In vivo* continuous-wave optical breast imaging enhanced with indocyanine green. Med. Phys..

[167] Owen-Reece H., Smith M., Elwell C.E., Goldstone J.C. (1999). Near infrared spectroscopy. Br. J. Anaesth..

[168] Villringer A., Planck J., Hock C., Schleinkofer L., Dirnagl U. (1993). Near infrared spectroscopy (NIRS): A new tool to study hemodynamic changes during activation of brain function in human adults. Neurosci. Lett..

[169] Custo A., Boas D.A., Tsuzuki D., Dan I., Mesquita R., Fischl B., Grimson W.E.L., Wells M. (2010). Anatomical atlas-guided diffuse optical tomography of brain activation. Neuroimage.

[170] Ntziachristos V., Ripoll J., Weissleder R. (2002). Would near-infrared fluorescence signals propagate through large human organs for clinical studies?. Opt. Lett..

